# Applications of Fast Iterative Filtering in NMR Spectroscopy: Baseline Correction

**DOI:** 10.1002/mrc.70004

**Published:** 2025-07-06

**Authors:** Letizia Fiorucci, Francesco Bruno, Marco Ricci, Antonio Cicone, Enrico Ravera

**Affiliations:** ^1^ Department of Chemistry “Ugo Schiff” Università degli Studi di Firenze Firenze Italy; ^2^ Magnetic Resonance Center Università degli Studi di Firenze Firenze Italy; ^3^ Consorzio Interuniversitario Risonanze Magnetiche di Metalloproteine Sesto Fiorentino Italy; ^4^ Dipartimento di Scienze e Innovazione Tecnologica Università del Piemonte Orientale Vercelli Italy; ^5^ Dipartimento di Ingegneria e Scienze dell'Informazione e Matematica Università degli Studi dell'Aquila L'Aquila Italy; ^6^ Istituto di Astrofisica e Planetologia Spaziali INFN Rome Italy; ^7^ Istituto Nazionale di Geofisica e Vulcanologia Rome Italy; ^8^ Florence Center For Data Science Università degli Studi di Firenze Firenze Italy

**Keywords:** baseline distortion, broadband excitation, pNMR

## Abstract

Fast iterative filtering (FIF) is a recently introduced signal decomposition technique related to empirical mode decomposition (EMD), which has been developed for the analysis of non‐stationary signals. When applied to the analysis of NMR data, FIF effectively partitions broad and narrow features by decomposing signals into intrinsic mode functions. In this work, we prove that FIF excels at separating baseline components from peaks, even in heavily distorted spectra. This capability is precious for processing spectra of paramagnetic compounds.

## Graphical abstract

Fast iterative filtering is a signal decomposition technique related to empirical mode decomposition. When decomposing NMR data into intrinsic mode functions, FIF effectively partitions broad baseline components from narrow peaks components, even in heavily distorted spectra, for instance, those of paramagnetic compounds.

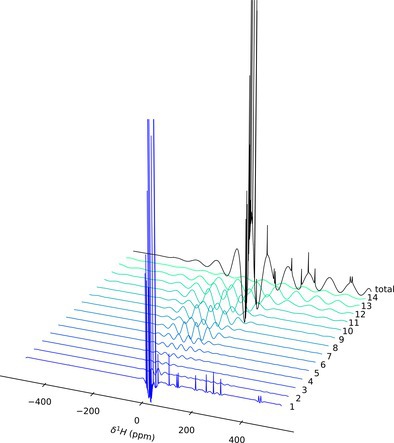

**Abbreviations**
CPR: curvature‐to‐power ratio
EMD: empirical mode decomposition
FFT: fast Fourier transform
FIF: fast iterative filtering
IMF: intrinsic mode functions
RMSD: root mean square deviation

## 1 Introduction

Empirical mode decomposition (EMD) is a signal decomposition technique used for nonstationary signals, introduced in a seminal paper by Huang and co‐workers in 1998 [1]. This method partitions the signal into well‐behaved amplitude and frequency modulation components called intrinsic mode functions (IMFs). EMD—and the methods that are derived from it—is largely used for nonlinear and nonstationary data analysis.

Some recent papers, by groups at the China University of Petroleum, have started exploring the use of EMD for noise reduction in nuclear magnetic resonance, in particular, for relaxometry data [2, 3]. However, the EMD algorithm incorporates several heuristic and ad hoc components that complicate the analysis of its behavior and make it difficult to establish convergence in advance. This implies, among other things, that it is impossible to guarantee a priori that the EMD method does not introduce unwanted oscillations in the decomposition [4] and errors at the boundaries of the signal [5]. Additionally, this method can face stability issues when dealing with noise, as demonstrated in [6].

For all these reasons, many alternative techniques to EMD have been proposed over the years. Among them, the only one that is based on iterations and hence does not require any a priori assumption on the signal under investigation is the so‐called iterative filtering (IF) method proposed by Lin and coworkers in 2009 [7], which computes the signal moving average as a point‐by‐point local weighted average.

Given a signal s(x),x∈ℝ, let L(s) be the operator that computes its moving average. If we consider the low pass Fokker–Plank filters w(t) defined in [8], the moving average of the signal s(x) can be computed via the convolution

$$L(s)(x)=\int_{-l}^{l}s(x+t)w(t)dt$$

which is equivalent to computing a weighted average of s at every point x, using as weighting function the filter w. If we define $s_{1}=s$ and the operator $F_{1,n}(s_{n})=s_{n}-L_{n}^{\left(1\right)}(s_{n})=s_{n+1}$, which captures the fluctuation part of $s_{n}$ by simply subtracting from $s_{n}$ its moving average $L_{n}^{\left(1\right)}(s_{n})$, then the first IMF is given by

(1) $$I_{1}=\underset{n\to \infty }{\lim}F_{1,n}(s_{n})=s_{n}-L_{n}^{\left(1\right)}(s_{n}),$$

where $L_{n}^{\left(1\right)}$ depends on the mask length $l_{n}$, which is the length of the filter at step n, and the superscript refers to the fact that we are extracting the first IMF. These iterations that we have so far described constitute the *Inner Loop stage* of IF, which allows for computing each individual IMF. Similarly, applying the operators F to the remainder signal $s-I_{1}$, we obtain $I_{2}$, the second IMF. By subsequent iterations, we obtain the k‐th IMF as $I_{k}=\lim_{n\to \infty }F_{k,n}\left(r_{n}\right)=r_{n+1}$, where $r_{1}=s-I_{1}-\ldots -I_{k-1}$. The IF method stops when $r=s-I_{1}-\ldots -I_{m},\;m\in \mathbb{N}$, becomes a trend signal, which means that the remainder r has at most one local maximum or minimum, and hence the signal is decomposed into $s(x)=\overset{m}{\underset{j=1}{\sum }}I_{j}(x)+r(x)$. This part of the code is called the *Outer Loop*, which allows IF to derive all the IMFs.

In [9], the authors proved that if the filters are chosen properly, it is possible to guarantee a priori the convergence of the method. This has opened the door to other theoretical results, like the fact that IF decompositions do not contain unwanted oscillations [4] and that it is possible to estimate and bind the errors introduced by IF nearby the boundaries in a decomposition [10]. The numerical advantages of this method are described in detail in reference [5].

A faster implementation based on Fast Fourier Transform (FFT) is called the Fast Iterative Filtering (FIF) method [9, 11]. The idea is to reformulate (1) using the well‐known property that convolution becomes a product in Fourier space. Equation (1) thus becomes

(2) $$I_{1}=\text{iFFT}\left({\left(1-\text{diag}\left(\text{FFT}(w)\right)\right)}^{N_{0}}\text{FFT}(s)\right)$$

where FFT and iFFT stand for fast Fourier transform and inverse fast Fourier transform, respectively, and $N_{0}$ is the number of iterations needed to compute the first IMF, based on a predefined stopping criterion. The rest of the algorithm remains the same. We note that the algorithmic complexity of FIF is primarily determined by the FFT calculation, which is the main bottleneck of the algorithm. As a result, the algorithmic complexity of FIF is O(nlog(n)) where n represents the length of the analyzed signal. Equation (2) allows us to substantially speed up the algorithm, which becomes hundreds of times faster than the original IF technique.

**FIGURE 1:**
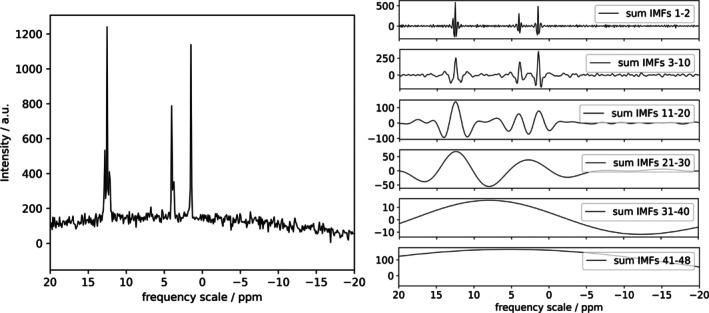
Decomposition of a simulated NMR signal using FIF algorithm. On the left, the original spectrum is presented, whereas the sum of different components is shown on the right. The IMFs are sorted from the fast‐oscillating ones to the slowest. This behavior enables the separation of different spectral features. The script for the data simulation and application of the decomposition algorithm is available as Listing 2.

The IMFs into which the signal is partitioned have different ranges of oscillations: on these grounds, we expect that FIF will be effective in separating narrow and broad features of the NMR spectrum (see Figure 1). It should be emphasized that no spectral features are lost or distorted during the decomposition process, in the sense that the sum of all IMFs produced by FIF for a certain dataset, like the one in Figure 1, is identical to the original spectrum (within numerical uncertainty).

Baseline distortions in NMR arise from various sources: (a) from a frequency‐dependent phase correction, which is a consequence of pulse imperfection [12] or of loss of the first few points of the FID due to the dead time (DE); (b) probe‐related properties, such as the glue in the electronic components which cause a characteristic gaussian‐like distortion [13, 14]. Typically, such baseline distorsions have a slow change across the spectrum [13]. Consequently, we expect that FIF will be more effective when signals exhibit significantly faster variations compared to the underlying background. This assumption is particularly reasonable in the context of solution NMR spectra.

In this paper, we demonstrate the effectiveness of FIF in removing common baseline distortions, typically broad features, from real experimental NMR spectra. These examples, representative of everyday solution NMR practice, highlight the practical utility of our approach.

## 2 Materials and Methods

### 2.1 Experimental Data

The experimental ^1^H spectra of dysprosium(III)‐DOTA (DyDOTA) [15] and nickel(II)‐Sal‐HDPT (NiSAL‐HDPT) [16, 17, 18] complexes were acquired, at room temperature, on a Bruker Avance NEO spectrometer operating at 1.2‐GHz ^1^H Larmor frequency with a 28.2‐T HTS/LTS hybrid magnet [19], using a 3‐mm triple resonance TCI cryo‐probehead (for NiSAL‐HDPT) and TXO cryo‐probehead (for DyDOTA). The experimental ^19^F spectrum of 3‐F‐tyrosine‐labelled human carbonic anhydrase II (3‐FY‐HCA II) [20] was recorded, at room temperature, on a Bruker Avance III HD spectrometer operating at 600‐MHz ^1^H Larmor frequency with a 14.1‐T superconductive magnet equipped with a 5‐mm single channel selective high power probe without gradient coil [21].

All the data can be found at https://zenodo.org/records/13796145.

### 2.2 Computational Details

The KLASSEZ library, available at https://github.com/MetallerTM/klassez, was used throughout the analysis for spectra simulation and processing of experimental data.

The FIF algorithm was applied using a dedicated Python implementation: https://github.com/EmanuelePapini/FIF. On a typical laptop, the average runtime to extract a single IMF from a 64K‐point spectrum is approximately 0.5 s. For spectra requiring multiple components, the total runtime may range from a few seconds to tens of seconds. The script for generating all the figures is given in the Supporting Information.

### 2.3 Evaluation of the Reconstruction Quality

The performance of FIF was quantitatively compared to other baseline correction methods on the benchmark set proposed by Schulze et al. [22]. The instructions for building the model are provided over a 1001 channels spectrum and will be here reported together with the instructions to build the model in the time domain. The model is built as follows: seven Lorentzian peaks are generated with peak centers at 340, 455, 432, 584, 618, 641, and 656 channels, respectively. All Lorentzians have FWHM of 5.7 channels, except for the peak at 340 channels that has a width of 10 channels. All peaks were convolved with a Gaussian of five channels width. In reference [22], the baseline is computed as

(3) $$b(x)=1+\frac{2}{\pi }\int_{0}^{x}e^{-t^{2}}dt$$

where x is the channel number, from 0 to 1000 and centered at 500. Besides a flat baseline, functions with midpoint slope (and correspondingly the maxima of the baseline) of 0.1, 0.3, and 1.0 are used. The peaks have all the same intensity, which is varied to achieve different signal‐to‐baseline ratios (SBR) of 0, 0.05, 0.1, 1.0, and 10. Also different levels of signal‐to‐noise ratio (SNR) are tested: 2, 3, 5, 10, and 100. Given the number of tests that are performed, the representation of the results is problematic, therefore, as figures of merit (FoM) the authors suggest the correlation coefficient between the estimated and the given baseline for the total spectrum, for the “uncongested” region (i.e., the region from 0 to 560 channels, where only three peasks are present) and for the “congested” region (i.e., the region from 561 to 1001 channels, where four peaks are present),

(4) $$r^{2}\left(Y,\hat{Y}\right)=\frac{SS_{reg}}{SS_{tot}}$$

with

(5) $$SS_{reg}=\sum_{i=n_{s}}^{n_{f}}{\left({\hat{Y}}_{i}-\overset{-}{Y}\right)}^{2}$$

and

(6) $$SS_{tot}=\sum_{i=n_{s}}^{n_{f}}{\left(Y_{i}-\overset{-}{Y}\right)}^{2}$$

where Y is the actual baseline, $\hat{Y}$ is the estimated baseline, $\overset{-}{Y}$ is the mean of Y, and $n_{s}$ and $n_{f}$ are the starting and final channels of the considered spectral region, that is, $n_{s}=0,\;n_{f}=560$ for the uncongested section and $n_{s}=561$ and $n_{f}=1000$ for the congested part. The $\chi^{2}$ between the estimated and the actual baseline is also computed, as a measure of the quality of the baseline subtraction.

(7) $$\chi^{2}=\sum_{i=0}^{1000}{\left({\hat{Y}}_{i}-Y_{i}\right)}^{2}$$

The channels and channel numbers were converted in ppm, assuming a starting spectral width of 12 ppm at 14.1 T. The noise was simulated with the sim.noisegen function of the KLASSEZ library, centered at a carrier frequency of 4.7 ppm, imposing a standard deviation of the noise to reproduce the proper SNR. The code for the generation of the figure is reported in Listing 1. The code was also opportunely modified to study the performance of the FIF algorithm not only for this asymmetric baseline distortion, but also in case of baseline rolling and for the asymmetric distortion after the application of a windowing function. The FoM were generated for 4 different spectral windows (SW). Besides the original 12 ppm window, the 30, 54, and 72 ppm spectral analogs were also studied. Independently from the SW, the same number of channels were considered in the computation of the statistical parameters, that is, 1001.

## 3 Results and Discussion

The decomposition of the signal and baseline is performed by dividing the IMFs into two groups. The first group, containing faster‐oscillating components, is assigned to the signal, while the second group, consisting of slower oscillating components, represents the baseline. The goal is to leave behind the smoothest possible baseline to determine the optimal number of IMFs to include in the signal. This process can be assisted by analyzing the behavior of a parameter describing the baseline properties. We have found that the curvature‐to‐power ratio (CPR) exhibits distinct discontinuities in the vicinity of the optimal number of IMFs when plotted against the number of IMFs excluded from the baseline reconstruction (or, equivalently, included in the signal reconstruction). The CPR definition is based on a procedure commonly done in the regularization of inverse transforms in several MR applications [23, 24], and it is defined as

(8) $$CPR=||b^{\prime \prime }||/||b|{|}^{2}$$

where b is the sum of the IMFs that are assigned to the baseline and *′′* denotes the second derivative. Plots of CPR computed on the reconstructed baseline as a function of the number of the excluded IMFs are provided in the Supporting Information for each reconstruction discussed in the following sections. The optimal number of IMFs can be automatically determined by identifying the point in the CPR plot that exhibits the largest difference to the preceding point (excluding from this evaluation all the non‐oscillating components). In cases of low signal‐to‐noise ratio or severe spectral congestion, the CPR curve may not display a clear transition point. In such situations, we recommend visual inspection of the CPR profile and, if needed, manual adjustment of the number of retained IMFs. The CPR curve itself serves as a useful diagnostic for identifying such problematic cases.

### 3.1 The “Pathological” Case of Paramagnetic NMR

Whoever attempted to acquire NMR spectra of paramagnetic species knows that baseline distortions arising from the combination of pulse imperfection, DE, and probe background represent a “pathological case” when dealing with large spectral windows, and they must be carefully treated. The usual approaches to baseline correction in paramagnetic systems are piecewise polynomial subtractions and spline interpolation. A part of the baseline distortions might be mitigated by backward prediction in the time domain, which can be performed either through linear prediction [24] or more sophisticated approaches [25, 26]—but not all cases are equally easy to treat.

**FIGURE 2:**
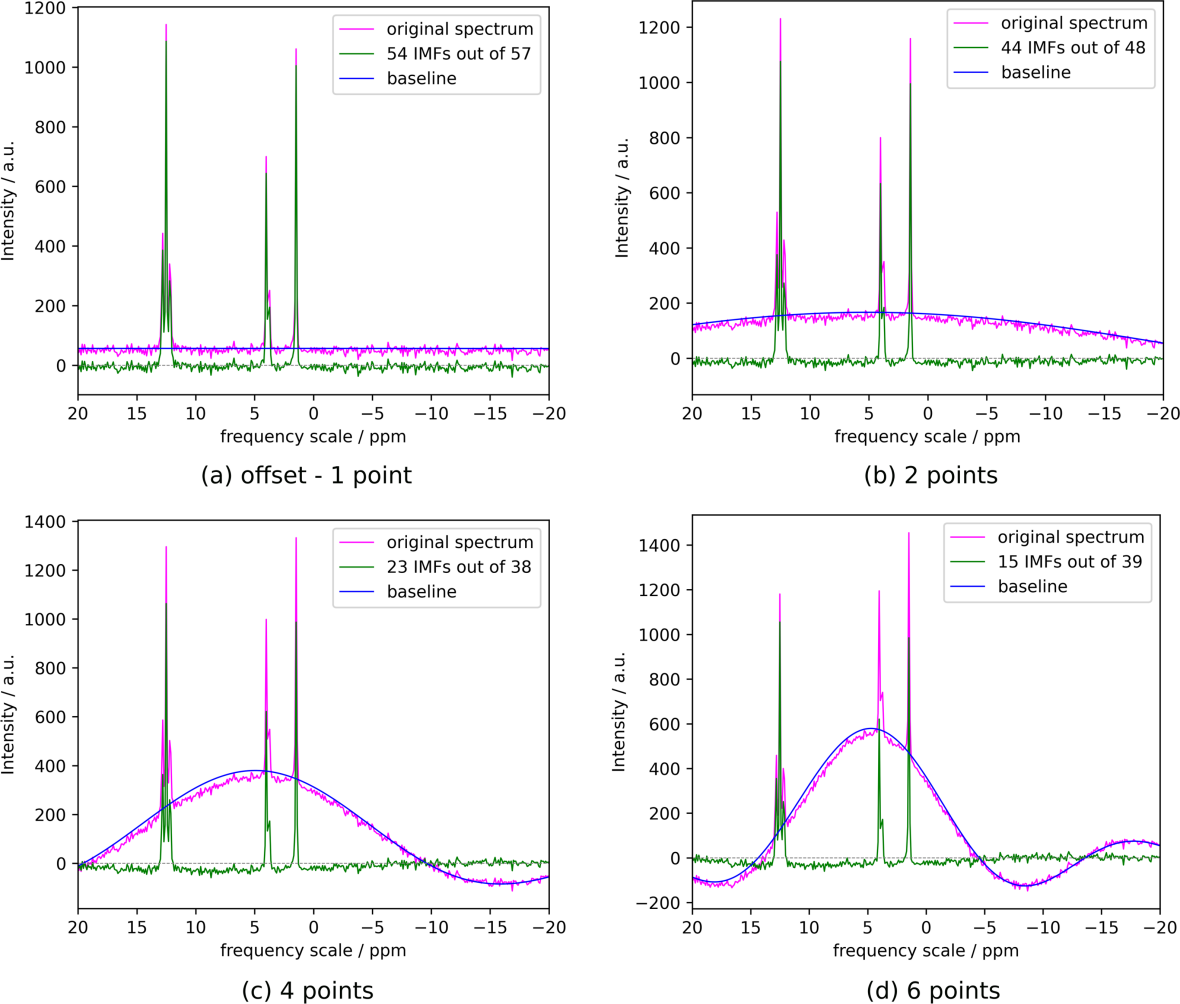
The efficiency of FIF for correcting wide baseline distortions is presented. The baseline oscillation effect is produced in the synthetic spectra through the intensity alteration of the first N points in the FID, over a wide spectral window. In general, the algorithm can considerably reduce the distortion, using a higher fraction of IMFs for larger alterations. A gray dashed line represents the ideal baseline. The Python code for the generation of this figure is presented in Listing 3.

To study the performance of FIF in correcting baseline distortions under these conditions, we tested the algorithm on synthetic FIDs with peaks of different linewidths and multiplicities and processed them after alteration of intensities for a certain number of points in the FID, mimicking the effect of the DE in a spectrum with large spectral window. This corresponds to adding a progressively more intense but narrower baseline distortion. The decomposition of such simulated spectra is shown in Figure 2. The number of IMFs included in the signal reconstruction is chosen according to the CPR criterium, as shown in Figure S2, where the optimal number of IMFs corresponds to the first discontinuity in the plot. Naturally, if the baseline is affected by an offset, this will be removed by excluding the only non‐oscillating component in the decomposition, that is, the last one. In general, the FIF decomposition is effective in removing the distortion and the number of IMFs used for baseline reconstruction is larger the longer the dead time, corresponding to faster and faster oscillations in the baseline‐IMFs.

The quality of the reconstruction shows a slight but nonlinear decline with increasing distortion, as evidenced by the plot of the RMSD (root mean square deviation) of the peak integrals calculated on the reconstructed spectrum compared to the original (non‐distorted) dataset (see Figure S1). Despite this, the quality of the correction remains satisfactory.

**FIGURE 3:**
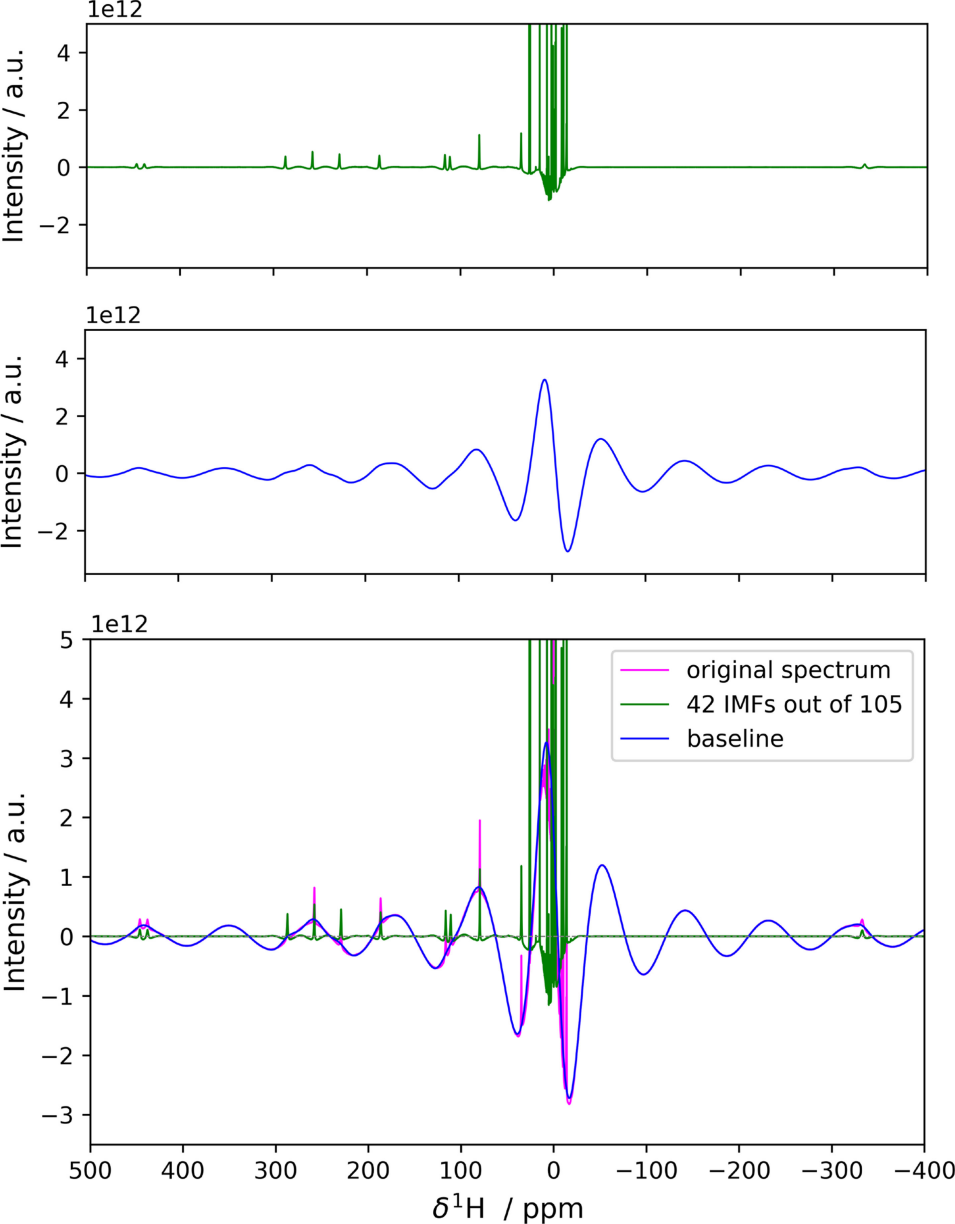
Application of FIF for baseline correction on NiSAL‐HDPT 1D ^1^H spectrum acquired at 1.2‐GHz ^1^H Larmor frequency. The code for the generation of this figure is reported in Listing 5. The corresponding CPR plot is reported in Figure S3.

A nice experimental example that collects several baseline issues in a single spectrum is provided by the archetypical paramagnetic complex (NiSAL‐HDPT), characterized by shifts over about 1000 ppm range in its ^1^H spectrum [17, 18], which make it amenable for testing high‐field instrumentation [14, 19]. High‐field spectra of Ni‐SAL‐HDPT often feature a significantly distorted baseline (Figure 3). Such a baseline distortion cannot be corrected automatically with the conventional methods, but requires either manual correction or the application of dedicated acquisition and processing schemes, as previously discussed [27]. Applying FIF and retaining 42 components significantly reduces the baseline and, although a manual correction (performed using Bruker TopSpin by manually adjusting the coefficients of a 4^th^‐degree polynomial baseline fit) affords a visually more satisfactory result, the relative intensities of the peaks are not dramatically altered (see Figures 3 and S4).

The impact of FIF processing on paramagnetic NMR spectra is even more apparent in the example of the ultra‐high magnetic field spectrum of a complex with a substantial magnetic anisotropy, where the field‐dependent Curie‐spin relaxation substantially broadens the signals [13, 28]. This scenario exacerbates the challenges described earlier, further complicating data analysis with the additional loss of intensity characteristic of these systems.

**FIGURE 4:**
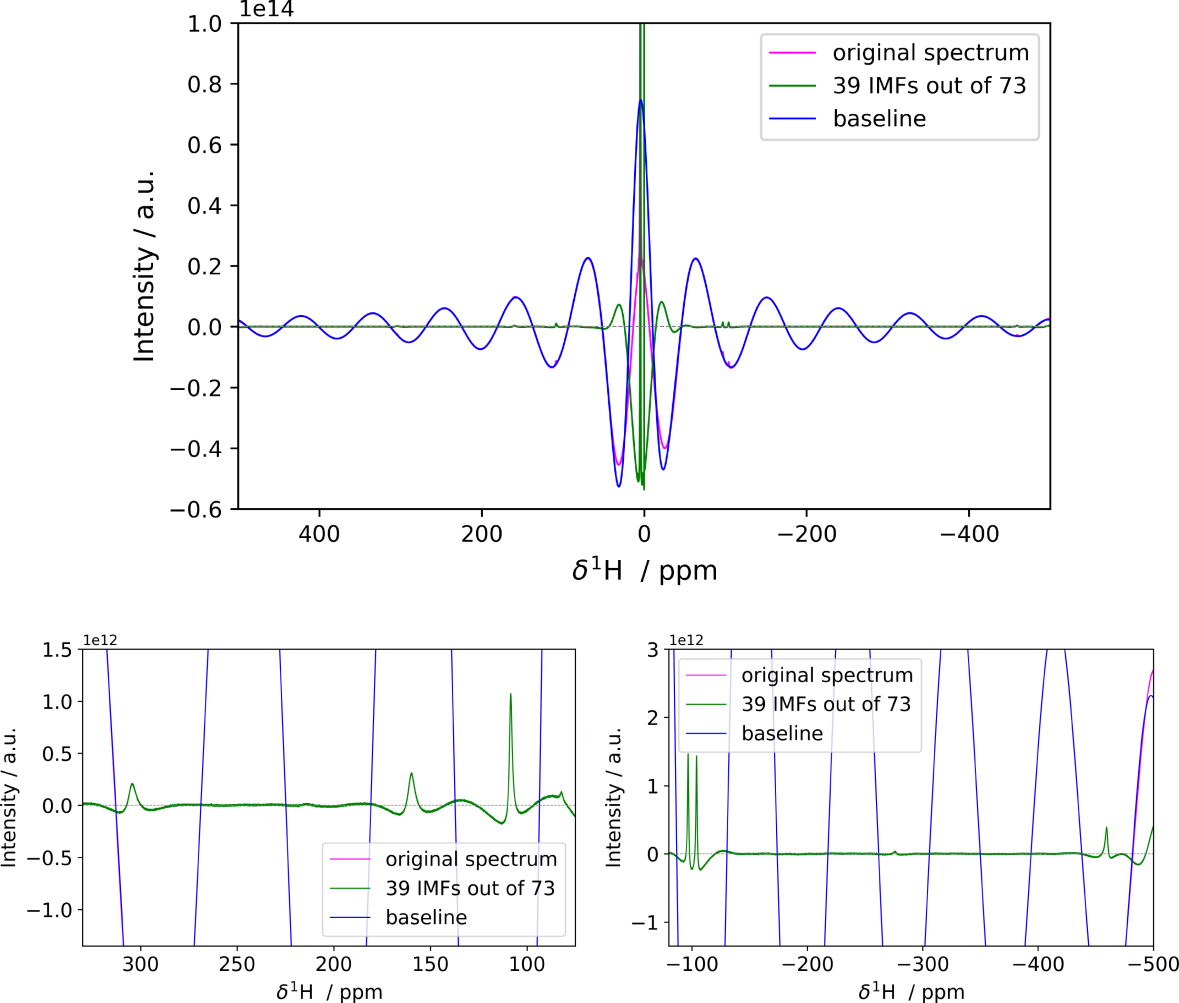
Application of FIF for baseline correction on dysprosium(III)‐DOTA 1D ^1^H spectrum acquired at 1.2‐GHz ^1^H Larmor frequency. A phase correction is applied before the application of the FIF algorithm. In the bottom panel, the spectra are magnified, to show the recovered peaks, which were undetectable both in phase‐distorted and phase‐corrected original spectra. The corresponding CPR plot is reported in Figure S5. The code for the generation of this figure is reported in Listing 5.

We selected the case of dysprosium(III)‐DOTA [15] ^1^H spectrum acquired at 28 T (see Figure 4) as an example for this scenario. Also in this case, FIF is found to provide a satisfactory separation of the baseline component in the spectrum (see Figure S5).

In the case of DOTA complexes, the spectra feature two sets of signals, corresponding to different diasteromers [29]. In the dysprosium(III) complex, the two pairs differ by 1 order of magnitude. As a further complication, the very large spectral window is associated with uneven excitation of the signals, so that at 1.2 GHz, the ratio between the most intense, and the less intense peak for the complex is around 30. Considering the solvent peak, the ratio exceeds five orders of magnitude. This makes this case ideal for testing the impact of a significant dynamic range on the reconstruction. The first observation is that the separation of slow and fast features allows the recovery of low‐intensity peaks in the paramagnetic regions, which is rather difficult to obtain in automation otherwise (see Figure S6).

A closer inspection of the reconstruction in the vicinity of the peaks reveals that the intensity of the peaks does not impact on the quality of the reconstruction (see bottom panels of Figure 4). The only parameter that appears to impact the FIF reconstruction of a peak is the difference between its linewidth and the “linewidth” of the baseline distortion. We have further investigated this point by a synthetic test of the following scenario: two peaks of equal linewidth, on top of a moderately distorted line, with the intensity of one peak which is progressively reduced with respect to the intensity of the second peak, which is held at the same level with respect to the noise. Since Lorentzian lines are very large at the base [30], it might be the case that the base of the peak stands significantly out of the noise. In such a case, broader IMFs will be needed to capture this behavior. This will make it difficult to select automatically the number of IMFs to include and will lead to an apparent overall worsening the reconstruction of the more intense peaks with respect to the reconstruction of the less intense peaks (see Figure S7). If we compare the trends of the reconstruction error for both peaks we can appreciate that the trends are identical, suggesting that the FIF algorithm performs equally well for peaks of different intensities (see Figure S8).

The successful application of FIF to both simulated and experimental data, including challenging cases with significant spectral broadening and complex baseline distortions, suggests that this algorithm can represent a robust and versatile tool for enhancing the quality and interpretability of paramagnetic NMR spectra.

### 3.2 Non‐Periodic Baseline Distortions

**FIGURE 5:**
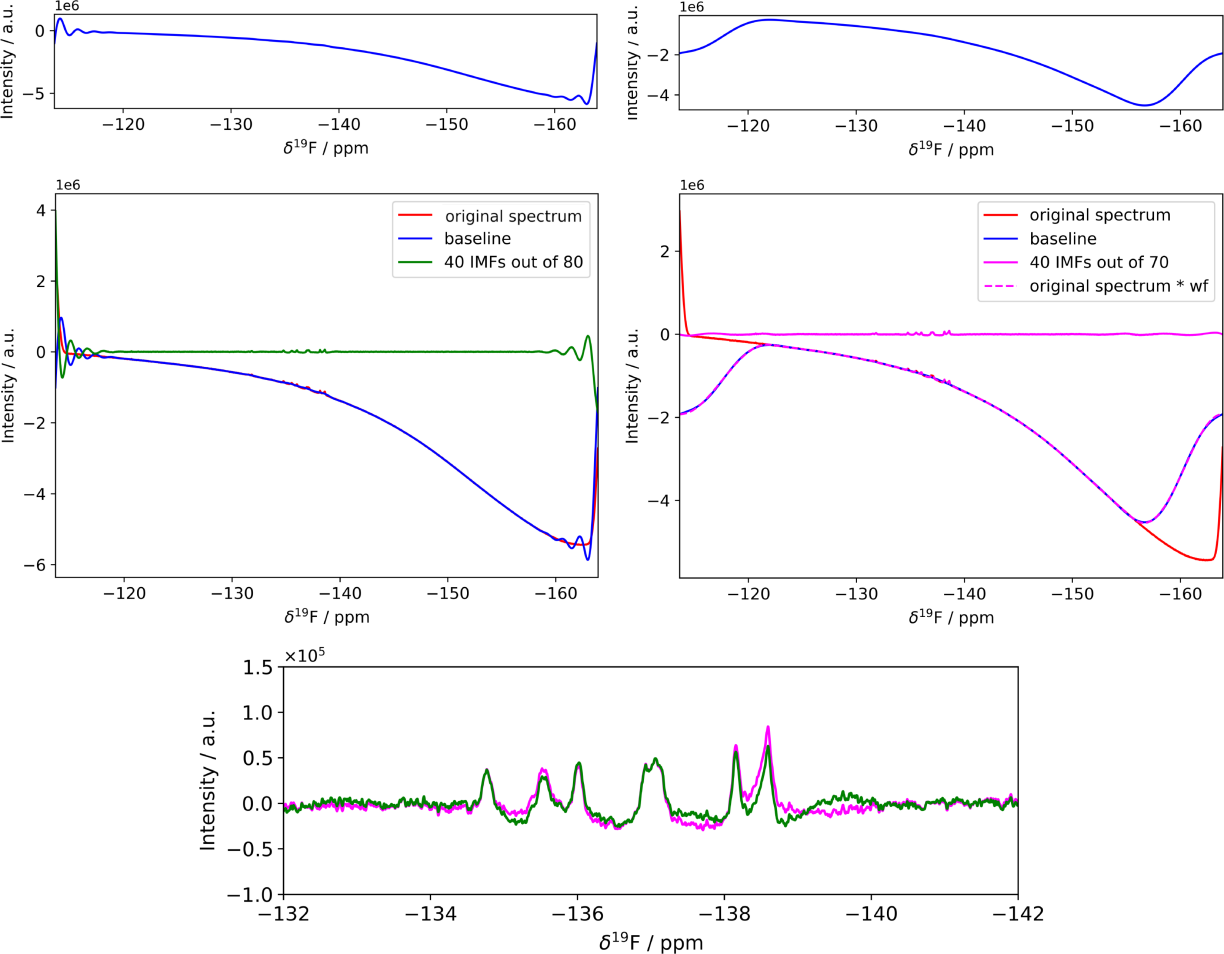
Application of FIF for baseline correction on ^19^F spectra of 3‐F‐tyrosine‐labelled human carbonic sample acquired at 600‐MHz ^1^H Larmor frequency. On the left the decomposition is performed on the untreated NMR data, while on the right, before FIF application, a sinusoidal window function is multiplied to the spectrum. The bottom panel makes a comparison of the signals reconstructed with and without the application of sinusoidal window function. The code for the generation of this figure is reported in Listing 6 and the routine for the application of the windowing function is in Listing 4.

While the FIF algorithm excels at decomposing signals with inherent periodicity, its effectiveness can be significantly impacted by non‐periodic features or localized baseline variations within the experimental data. Preprocessing techniques can be employed to address these limitations and improve the accuracy of signal reconstruction, and in particular, the spectrum can be made periodic at boundaries by applying a windowing [10]. As an example we have selected the probe‐induced broad features that appear in a ^19^F spectrum (Figure 5). The spectrum is made periodic at the boundaries as described in [10]. This makes the partitioning of the signals and baseline more efficient, as testified by the higher quality of the reconstructed signal after the application of this function (see Figure 5) and the more clear discontinuity observable in the corresponding CPR plot computed in the peaks region (see Figure S12). It is also apparent that the application of the windowing function prevents substantial variation in the relative intensities of the peaks in the reconstruction.

This successful application demonstrates the versatility of the FIF algorithm beyond challenging paramagnetic NMR cases. By incorporating tailored preprocessing steps, for example, the multiplication by an oscillating function, FIF can be adapted to effectively address various spectroscopic scenarios, including those with non‐periodic baseline distortions, thus establishing its potential as a general‐purpose tool for baseline correction.

### 3.3 The Phase Problem

In the discussion above, the decomposition procedure was applied exclusively to the real part of the NMR signal. Addressing the treatment of experimental spectra as complex arrays falls outside the scope of this manuscript and will be explored in future work. Nonetheless, it is worth examining the role of phase distortion in FIF decomposition of a NMR spectrum.

In principle, FIF does not need the spectra to be phased before baseline subtraction. However, the reconstruction of non‐phased signals is less efficient than the reconstruction of purely absorption peaks, as testified by the CPR plots constructed for the Ni‐SAL‐HDPT example, where the discontinuity is barely or not visible in this case. This is in line with what we observed in the ^19^F spectrum before and after the application of a periodic correction function, and reflects the difficulty of FIF in clearly separating the baseline components from the peaks in the presence of a non‐periodic distortion. Even if it has been designed for real data, the baseline correction for the imaginary channel can be obtained analogously to the real part through FIF decomposition. Interestingly, the CPR plot of the decomposition of the imaginary part of the not‐phased signal is more informative than the corresponding plot done on the real part of the data (see Figures S9 and S10), consequently, also the reconstruction of the latter is worse (see Figure S11).

Alternatively, the complex baseline signal can be recovered directly as the Hilbert transform [24] of the baseline correction for the real channel. This latter procedure leads to a reconstructed complex signal that, once phase‐corrected, results less distorted than the corresponding signal where both real and imaginary part were treated using FIF (see Figure S11).

### 3.4 A Systematic Estimation of the FIF Performance

Baseline subtraction is a particularly relevant issue in spectroscopy, consequently, countless baseline subtraction methods have been proposed for NMR and for other spectroscopies. A very interesting comparison of the performances of the different methods has been provided by Schulze et al. in [22]. In this work, the authors provide a benchmark set of signals and a baseline distortion model, and criteria for the evaluation of the performance of different baseline subtraction methods, together with a benchmark spectrum (for the description of the benchmark, see Section 2.3). We have applied the FIF algorithm to the benchmark spectrum, to provide FoM that can be directly compared to the results presented in [22]. As mentioned above, the benchmark spectrum has a non‐periodic baseline distortion, therefore we also extended the spectral window to 30, 54, and 72 ppm. The rationale behind the study of multiple SWs is that the FIF algorithm becomes more efficient the more the baseline features can be distinguished from the peaks based on their oscillation frequency, therefore, an enhancement of the oscillation properties of the baseline should be beneficial in this sense. This echoes what was observed in [22], where the authors found a relatively poorer performance for methods such as wavelet decomposition. However, the improvement of the reconstruction with increased spectral width is not monotonic. The enlargement of the spectral features helps in the spectra reconstruction up to the point where the distortion starts to look “narrow” compared to the entire window. This limit is reproduced by keeping the linewidth of the sigmoidal baseline constant and independent from the spectral width of the synthetic spectra. This of course does not apply to the Slope 0 baseline, whose reconstruction improves indefinitely with the increase of the spectral width (see Figures S13–S16).

Another interesting feature to notice is that the reconstruction improves with a lower the SNR. This can seem counterintuitive at first. Our interpretation is that, since FIF works by similarities in the oscillation properties, an increased level of noise ‐ i.e. an increased presence of random frequencies generally faster than baseline oscillations and more similar to the peaks oscillations ‐ can help the algorithm to better discern the two features.

Given that the benchmark set has a non‐periodic baseline, which represents an intrinsic problem for FIF we added a windowing function as done for the ^19^F spectrum. When windowing was applied on top of the baseline distortion, the linewidth of the sigmoid function was kept equal to the previous case, whereas the window function was broadened in order to reproduce the same oscillatory features independently of the spectral width. We provide an additional set of FoM‐evaluated on the reconstructed spectrum rather than on the reconstructed baseline, because a windowed baseline is reconstructed instead. Comparing Figures S13–S16 with Figures S21–S24 and Figures S17–S20 with Figures S25–S28, we observe that, on average, the windowing function improves the reconstruction of spectral features across all SBR, SNR, and Slope values with the exception of the Slope 0 baseline. In that case, no improvement is observed, as the windowing function is not applied. Within the same series, we can see similar trends to the one described for the situation without windowing, so also in this case lower SNR values and wider spectral windows help in the reconstruction.

As a final test, the benchmark spectrum is modified with the periodic baseline distortion arising from dead time. The results are shown in Figures S29–S32 and S33–S36. In a more apparent way with respect to the previous cases, the SW increase consistently improves the reconstruction.

Tables 1 and 2 collect the average $\chi^{2}$ and correlation coefficient $r^{2}$ for the reconstructed baseline for the different types of baseline distortion investigated with this method: asymmetric baseline distortion (Asymm.), asymmetric baseline distortion with windowing function (Asymm. wf.) and baseline rolling (Rolling). The average is performed over the possible SNR, SBR, and Slope or N. points values. As anticipated, the average $\chi^{2}$ is lower for the rolling baseline, while the correlation coefficient is higher. This is due to the fact that the rolling baseline is more similar to a pure sine function, which is easier to reconstruct and separate from the peaks features. The average $\chi^{2}$ decreases with increasing spectral width. Comparison of Asymm. and Asymm. wf. shows that the windowing function improves the reconstruction for small spectral widths, while it is not effective for larger spectral widths, for which the application of the FIF algorithm without the windowing function pre‐treatment is already effective for a successful spectra reconstruction. The accuracy of the FIF algorithm as evaluated from the $\chi^{2}$ compared to the values reported in Figure 13 of reference [22] is quite good, comparable to (e.g.) the average performance of the Wavelet method ( ∼660) and somewhat worse than Noise Median method ( ∼340), evaluated over the same spectral width. Using the correlation coefficient of the reconstructed baseline as a FoM, the performance of FIF appears to be comparable to the one of the Maximum Entropy Reconstruction method, ( ∼0.2), which is particularly poor. However, if the correlation coefficient is calculated on the reconstructed spectrum (Table 3), the performance of FIF is comparable to that of all the other methods.

**TABLE 1:** Average $\chi^{2}$ for the different types of baseline distortion investigated with the method described in Section 2.3: asymmetric baseline distortion (Asymm.), asymmetric baseline distortion with windowing function (Asymm. wf.), and baseline rolling (rolling).

Spectral width (ppm)	Asymm.	Asymm. wf.	Rolling
12	574	597	332
30	338	328	219
54	236	231	165
72	111	107	132

*Note:* The average is performed over the possible SNR, SBR, and slope or N. points values. Each value refers to a certain SW value, reported in the first column of the table in ppm.

**TABLE 2:** Average correlation coefficient for the different types of baseline distortion investigated with the method described in Section 2.3: asymmetric baseline distortion (Asymm.), asymmetric baseline distortion with windowing function (Asymm. wf.), and baseline rolling (rolling).

Spectral width (ppm)	Asymm.	Asymm. wf.	Rolling
12	0.16	0.08	0.20
30	−0.37	−0.61	−0.60
54	0.23	−0.15	0.24
72	0.05	0.02	0.19

*Note:* The average is performed over the possible SNR, SBR, and Slope or N. points values. Each value refers to a certain SW value, reported in the first column of the table in ppm.

**TABLE 3:** Average correlation coefficient for the signals for different types of baseline distortion investigated with the method described in Section 2.3: asymmetric baseline distortion (Asymm.), asymmetric baseline distortion with windowing function (Asymm. wf.), and baseline rolling (rolling).

Spectral width (ppm)	Asymm.	Asymm. wf.	Rolling
12	0.36	−0.20	−0.10
30	0.58	0.44	0.36
54	0.59	0.51	0.46
72	0.56	0.62	0.46

*Note:* The average is performed over the possible SNR, SBR, and Slope or N. points values. Each value refers to a certain SW value, reported in the first column of the table in ppm.

## 4 Conclusions

This study demonstrates the potential of fast iterative filtering (FIF) as a tool for addressing baseline distortions in NMR spectroscopy. By effectively separating broad baseline features from narrow spectral peaks, FIF showed promise in handling challenging cases, including heavily distorted spectra of paramagnetic substances. This capability contributes to improved peak recovery and enhanced interpretability of NMR data.

While FIF performs reliably across a broad range of resolutions typical for NMR spectra, its performance can be limited in spectra with extreme peak crowding, very limited baseline regions, or broad peaks that resemble the baseline in frequency scale. Such cases may require careful manual adjustment or alternative correction strategies.

Although not claiming to represent the full diversity of NMR spectral distortions, we have shown that FIF can be successfully applied to NMR spectra for baseline subtraction, including cases with non‐periodic baseline characteristics, and has a behavior similar to other methods. Notably, this work represents the first application of FIF to NMR spectroscopy.

Considering its nearly negligible computational cost, the results obtained using FIF are encouraging. This initial exploration suggests that FIF offers a computationally efficient and adaptable approach to baseline correction in NMR. We anticipate that this initial application will stimulate further development and broader applications of FIF in NMR spectroscopy.

## Conflicts of Interest

The authors declare no conflicts of interest.

## Peer Review

The peer review history for this article is available at https://www.webofscience.com/api/gateway/wos/peer‐review/10.1002/mrc.70004.

## Supporting information



Supporting_information_FIF.pdf

## Data Availability

The data that support the findings of this study are openly available in 13796145 at https://zenodo.org, reference number 13796145.
