# Supplementary material for: Applications of Fast Iterative Filtering in NMR Spectroscopy: Baseline Correction
Source: Magn Reson Chem. 2025 Jul 6;63(9):737–47. doi: 10.1002/mrc.70004 (PMC12318907; doi:10.1002/mrc.70004)
Supplement: Supplementary file 1 — Supporting_information_FIF.pdf [file MRC-63-737-s001.pdf]

Applications of Fast Iterative Filtering in NMR spectroscopy: Baseline correction

S1 | CODE SPECIFICATIONS

The analysis presented in the manuscript has been performed in a miniconda environment with Python v3.12.5 and FIF v2.13, available at https://github.com/EmanuelePapini/FIF. The additional dependencies are: `numpy` v2.0.2, `matplotlib` v3.9.2, `nmrglue` v0.10 and `KLASSEZ` v0.4a.5.

The synthetic spectra in Figures 1 and 2 of the main text were generated using `KLASSEZ` with the following specifications:

```
B0 14.1
nuc 1H
o1p 4.7
SWp 100
TD 2**10
shifts 4, 3.77, 1.47, 12.5
fwhm 50.0, 50.0, 50.0, 50.0
amplitudes 2, 1, 3, 6
x_g 0, 0, 0, 0
mult s, s, s, t
Jconst 0, 0, 0, 200
```

The noise was added using the `sim.noisegen(size, o2, t2, sn)` routine, which simulates additive noise in the time domain in the form of a matrix of dimensions `size`. This model for the noise depicts it as a white noise vector, modulated for the carrier frequency o_2 [S31]. The input parameters are: `size`: dimensions of the final noise matrix, `o2`: carrier frequency, `t2`: timescale of the last temporal dimension, `sn`: standard deviation of the simulated noise.

S2 | ADDITIONAL PLOTS

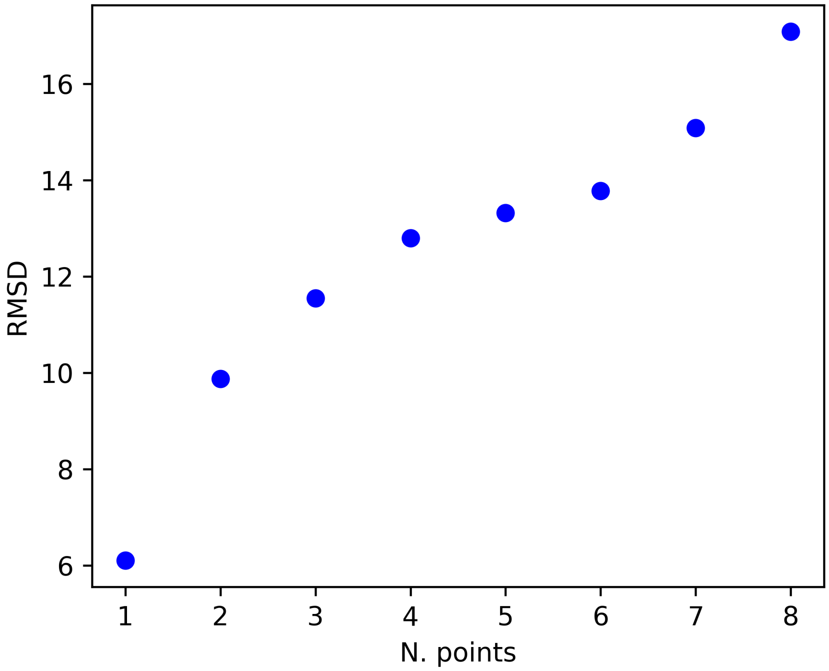

FIGURE S1 RMSD values computed between the reconstructed spectrum and the corresponding synthetic (not-distorted) spectrum, for different degrees of baseline distortion, represented as the number of points of the FID altered in intensity (see Figure 2).

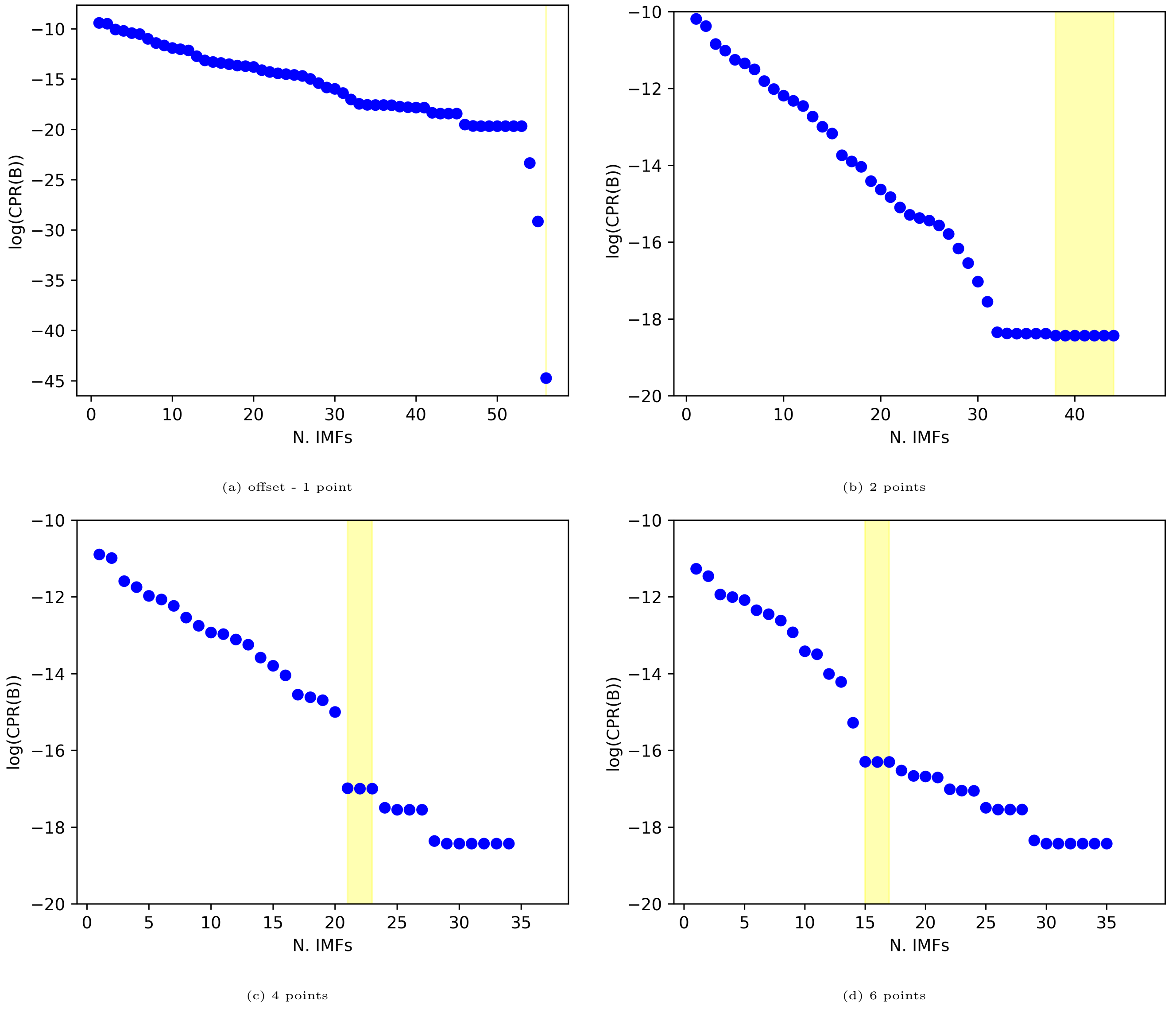

FIGURE S2 Plot of CPR logarithm computed on the baseline as function of the number of IMFs excluded from its reconstruction, for the synthetic spectra presented in Figure 2. The yellow region indicates the optimal number of IMFs that can be included in the signal, which leads to the reconstructed spectrum of Figure 2. In panel (b), even if the CPR criteria would have selected the IMFs from 32 on, we manually switched to the following set to slightly improve the reconstruction, but the difference is subtle.

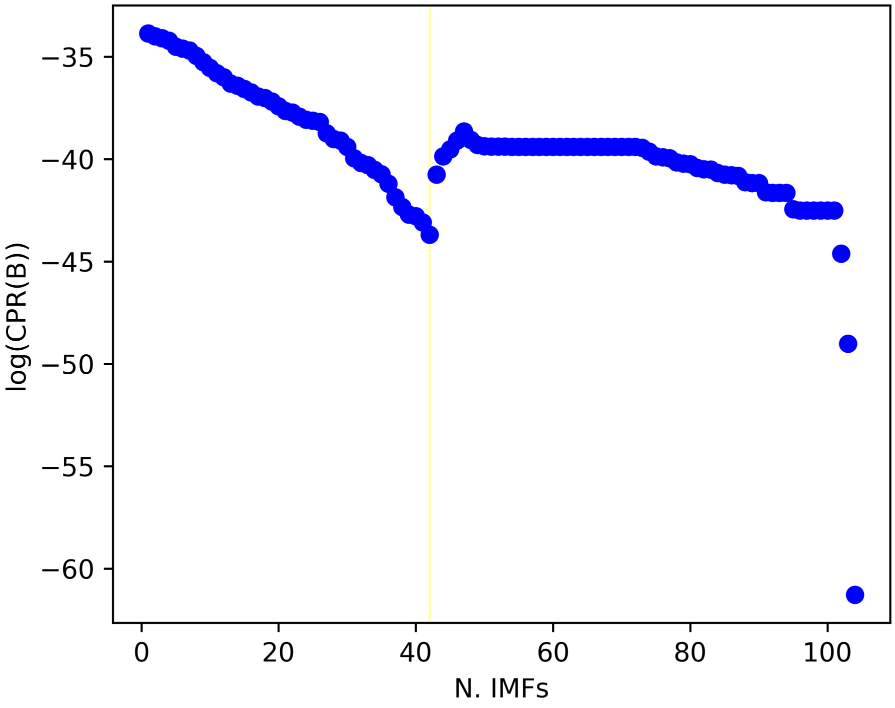

FIGURE S3 Plot of CPR logarithm computed on the baseline as function of the number of IMFs excluded from its reconstruction, for the experimental spectrum of Figure 3. The yellow line indicates the optimal number of IMFs that can be included in the signal, which leads to the reconstructed spectrum of Figure 3.

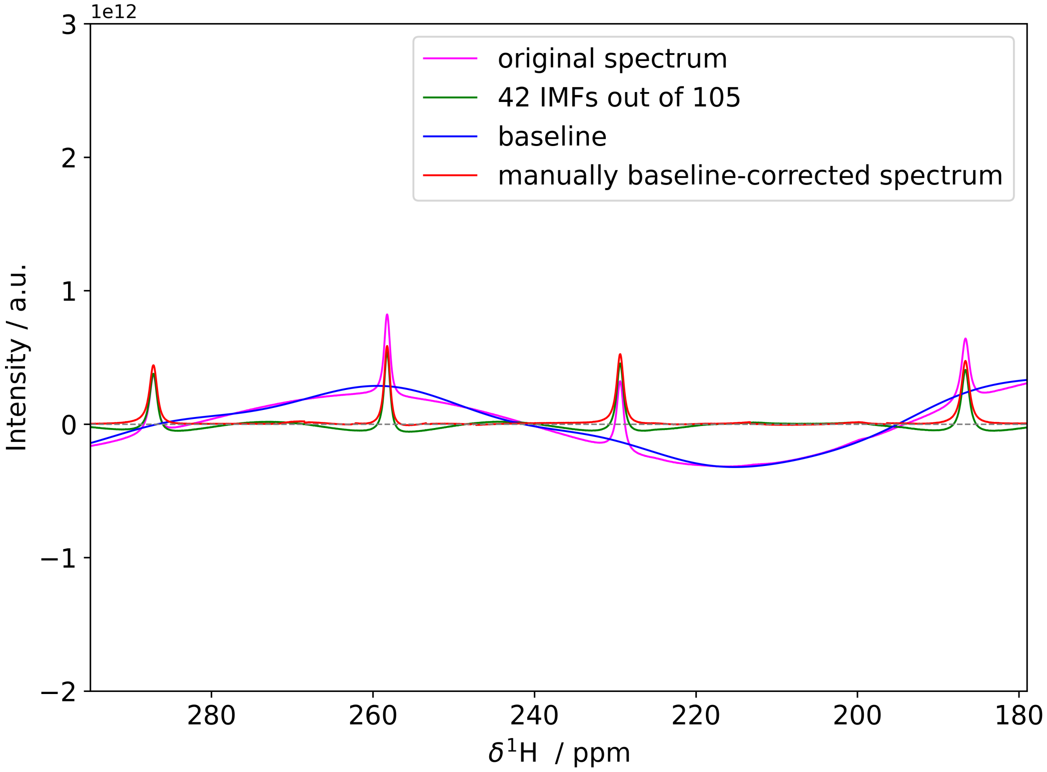

FIGURE S4 Section of the Ni-SAL-HDPT 1D ^{1}H spectrum acquired at 1.2 GHz ^{1}H Larmor frequency decomposed with FIF, from Figure 3. The comparison is also made with the manually corrected version of the experimental spectrum.

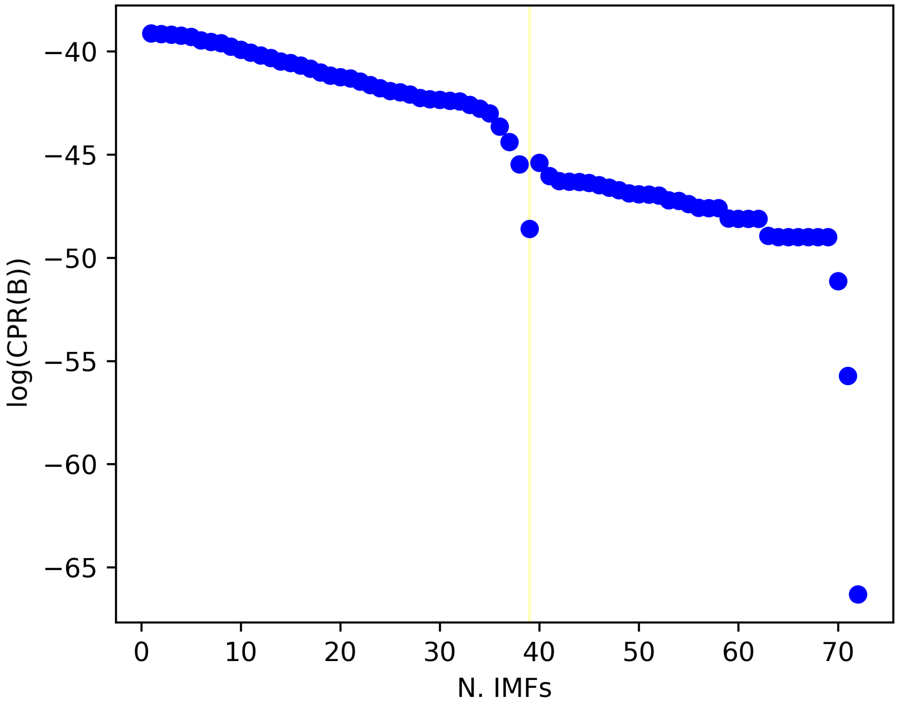

F I G U R E S5 Plot of CPR logarithm computed on the baseline as function of the number of IMFs excluded from its reconstruction, for the experimental spectrum of Figure 4. The yellow line indicates the optimal number of IMFs that can be included in the signal, which leads to the reconstructed spectrum of Figure 4.

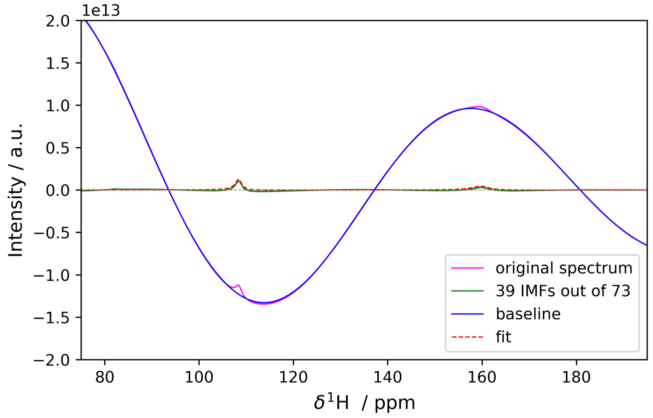

F I G U R E S6 Comparison of baseline correction performed with FIF and with fitting of the signal lineshape.

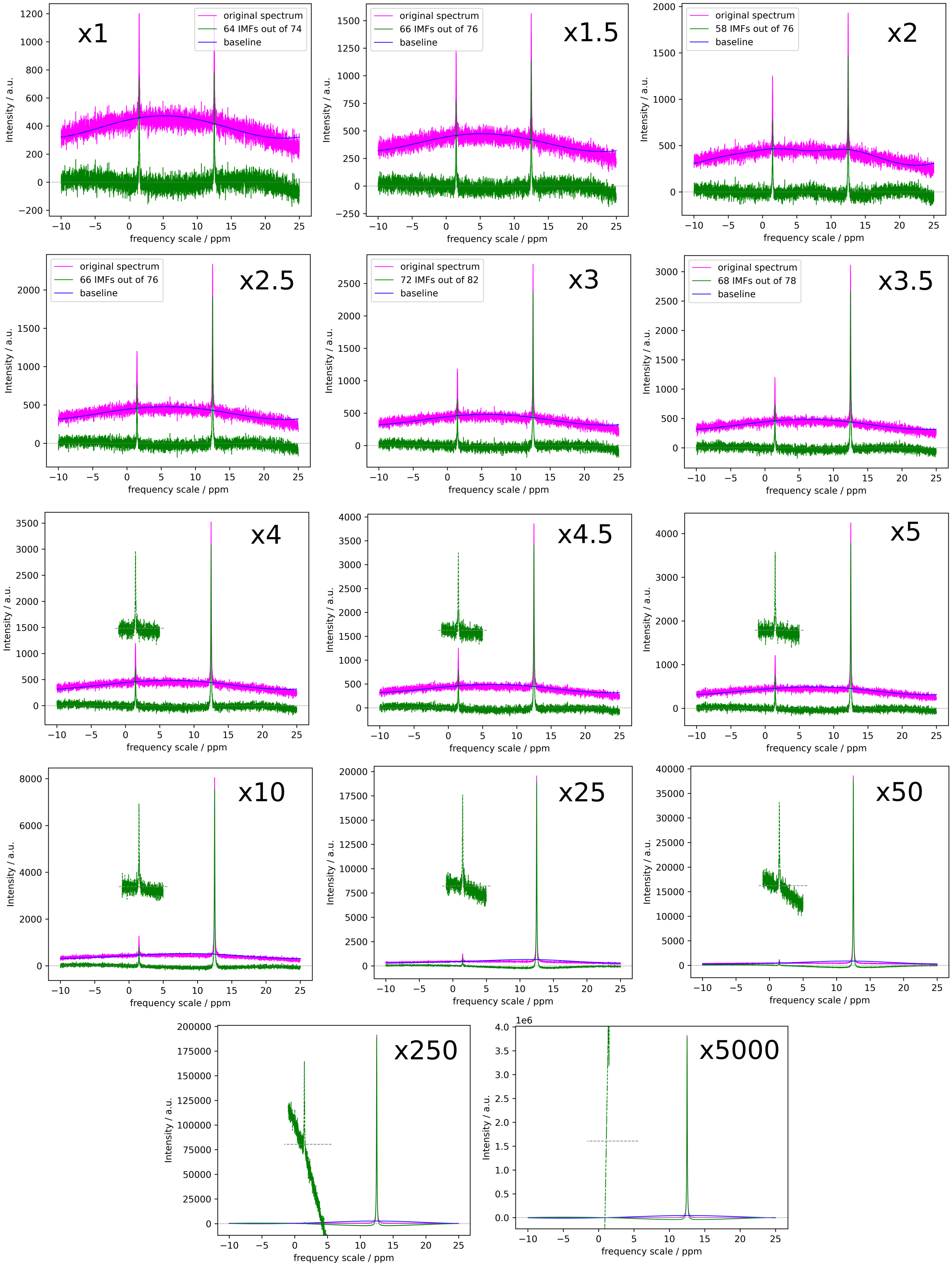

FIGURE S7 Baseline correction with FIF algorithm performed on simulated spectra with increasing dynamic range.

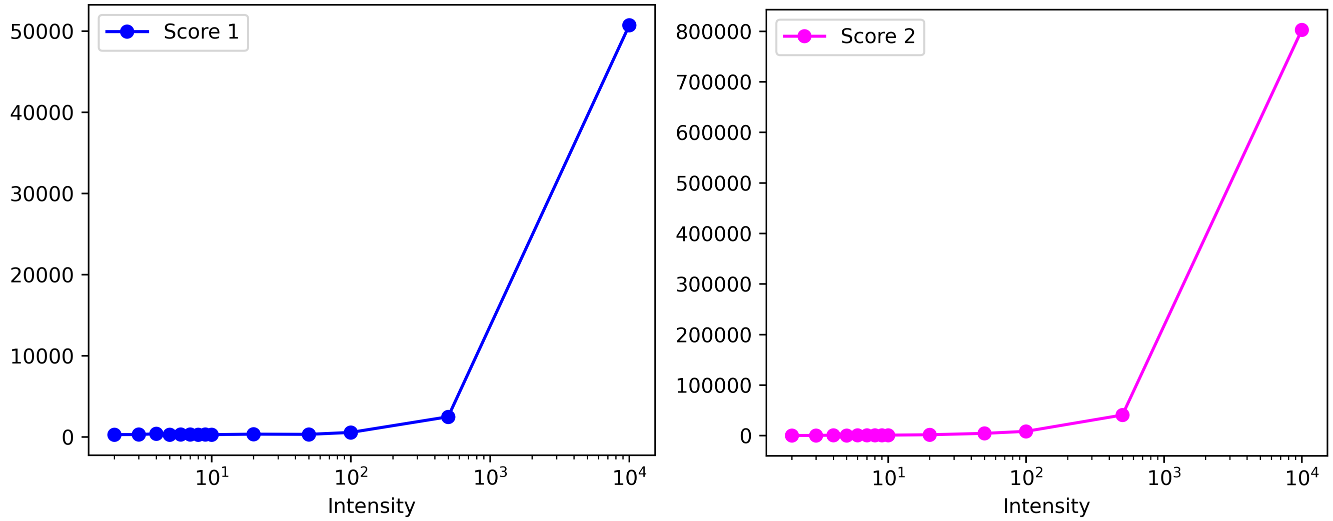

FIGURE S8 Score computed for the reconstruction of smaller signal (score 1) and bigger signal (score 2) as a function of the intensity of the second peak. The score is computed as the RMSD between the original -not baseline distorted- spectra and the reconstructed one, considering the same range of points around each peak.

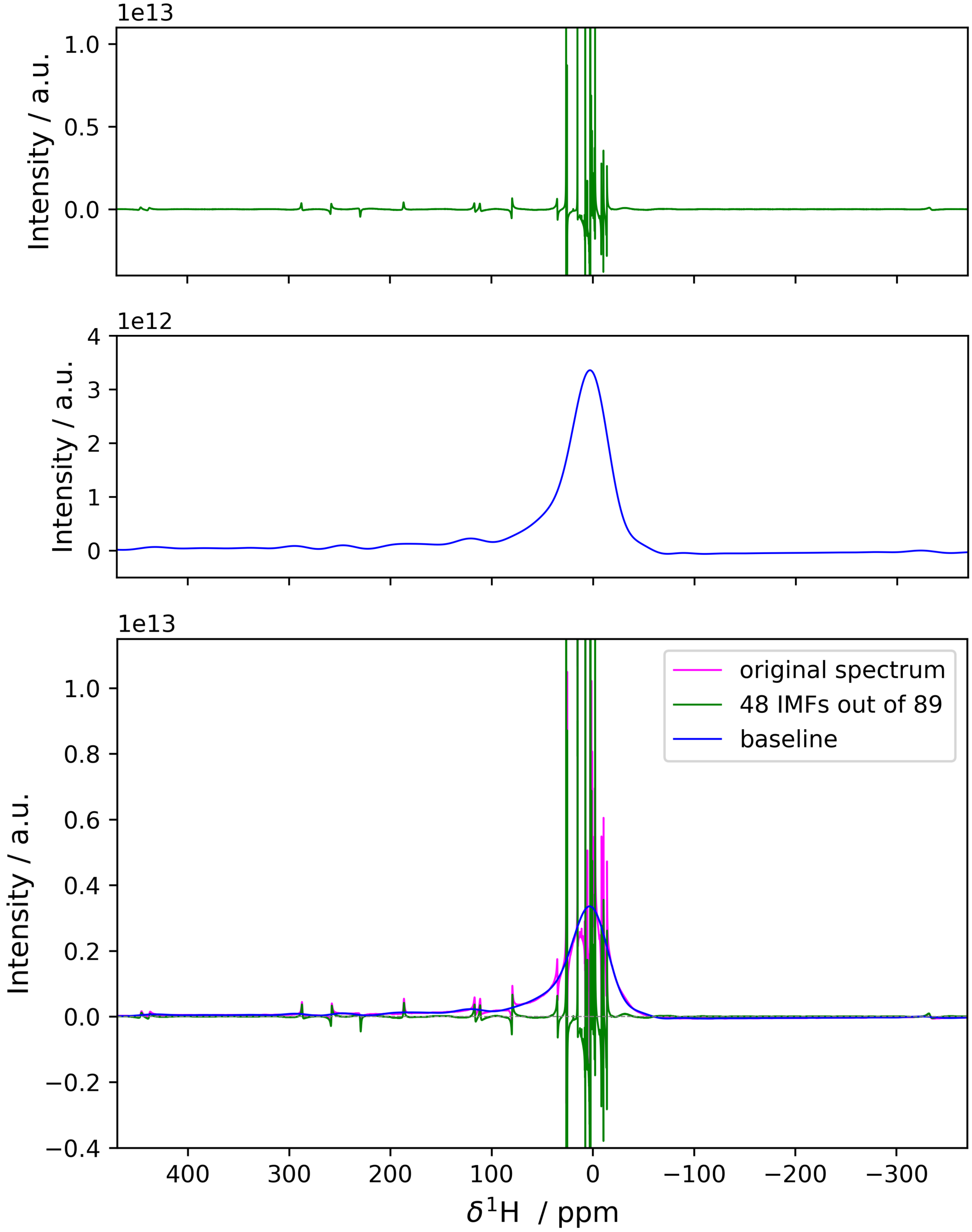

FIGURE S9 FIF decomposition and reconstruction of the real part of the not-phased version of the Ni-SAL-HDPT experimental spectrum of Figure 3.

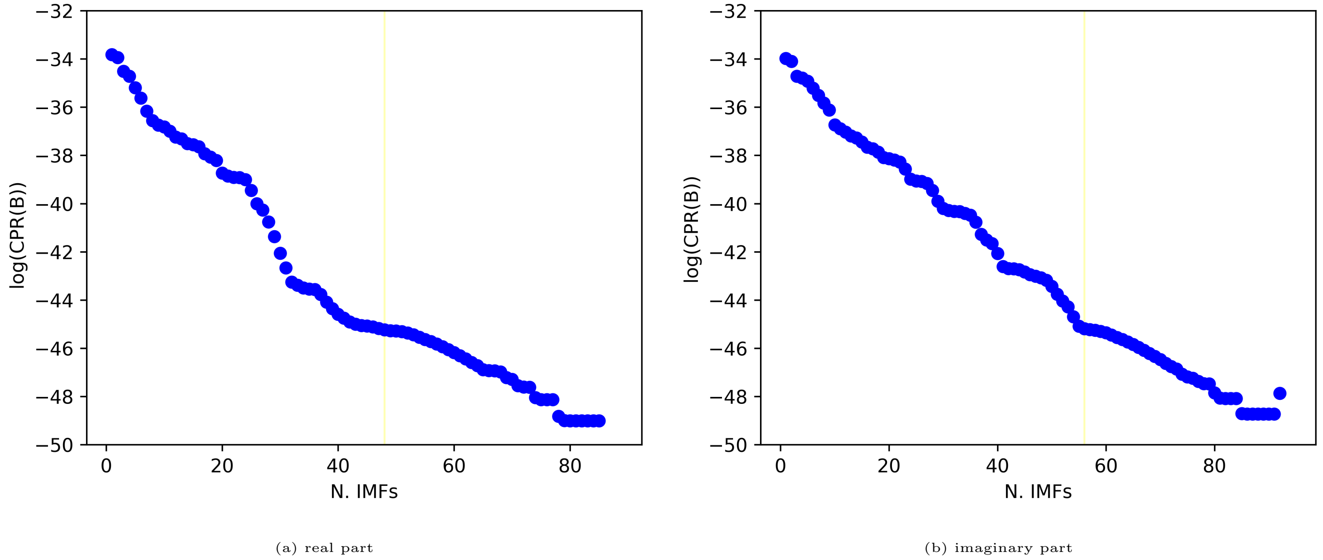

FIGURE S10 Plot of CPR logarithm computed on the baseline as function of the number of IMFs excluded from its reconstruction for the not-phased Ni-SAL-HDPT experimental spectrum. The yellow line indicates the optimal number of IMFs that can be included in the signal, which leads to the reconstructed spectrum of Figure S11. The comparison of these plots with the one in Figure S3 highlights the higher quality of the decomposition obtained after phase correction of the spectrum. Additionally, among the two, the one that is decomposed the best is the imaginary part.

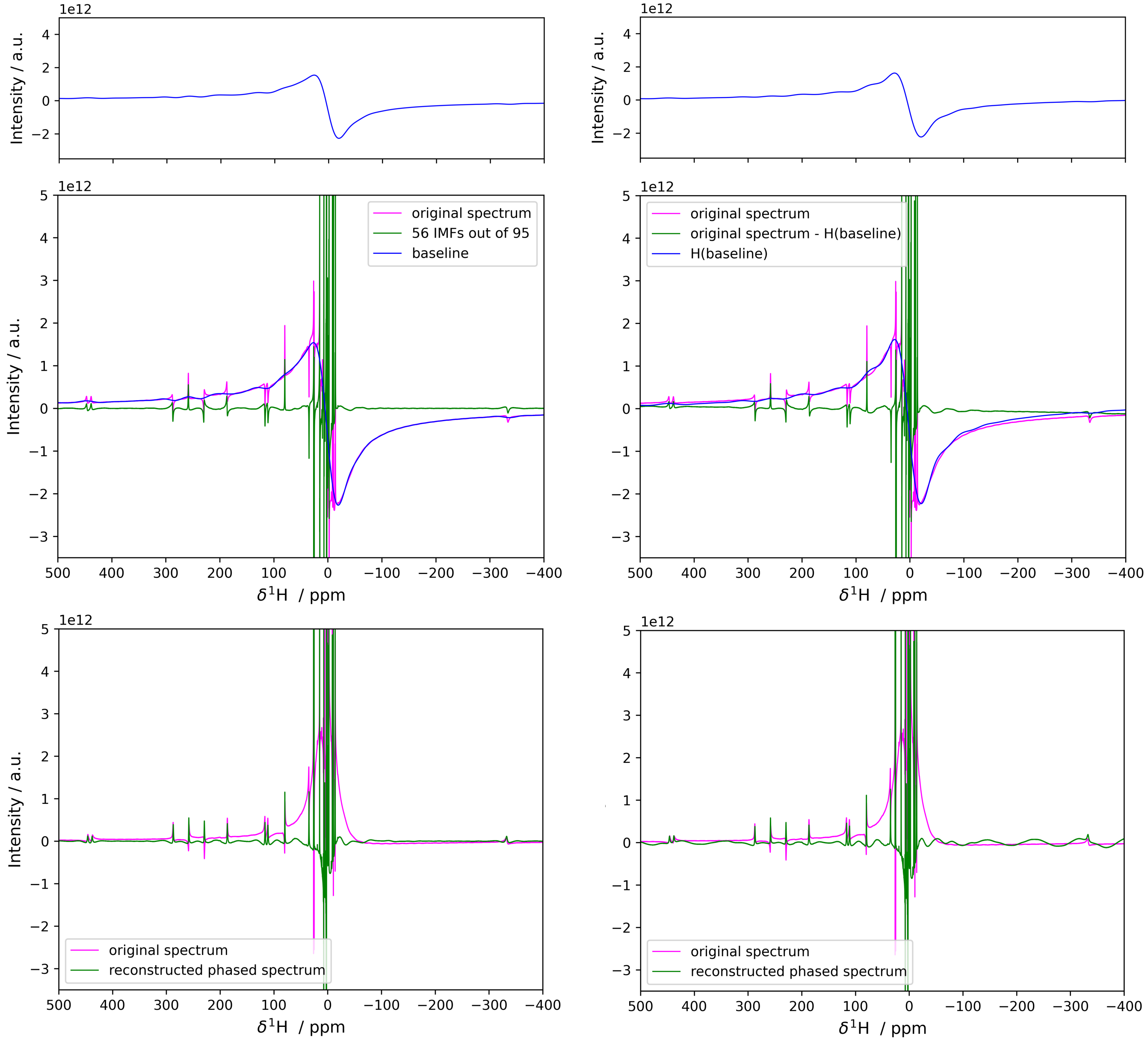

FIGURE S11 FIF decomposition and reconstruction of the imaginary part of the not-phased version of the Ni-SAL-HDPT experimental spectrum of Figure 3. It is presented either as the sum of IMFs from direct decomposition of the imaginary part of the spectrum (left) or from the Hilbert transform of the baseline from Figure S9 and subtraction to the imaginary part of the experimental spectrum (right). The bottom panels compare the non-phased real spectrum and the real part of the phased spectrum composed by the sum of the reconstructed real (from Figure S9) and imaginary (from the corresponding plots above) spectra.

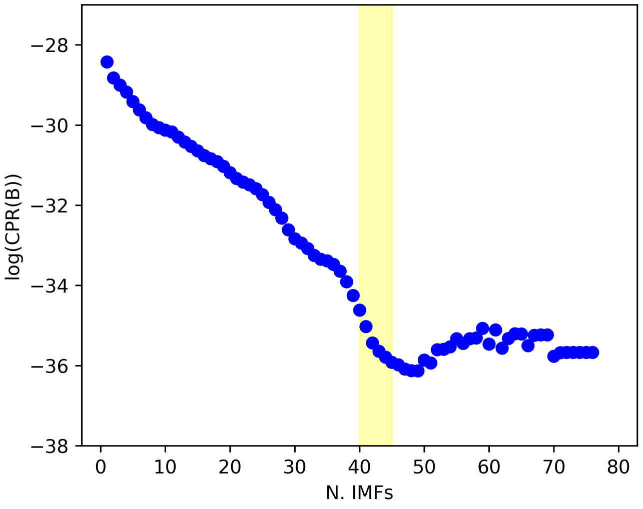

(a) without window function

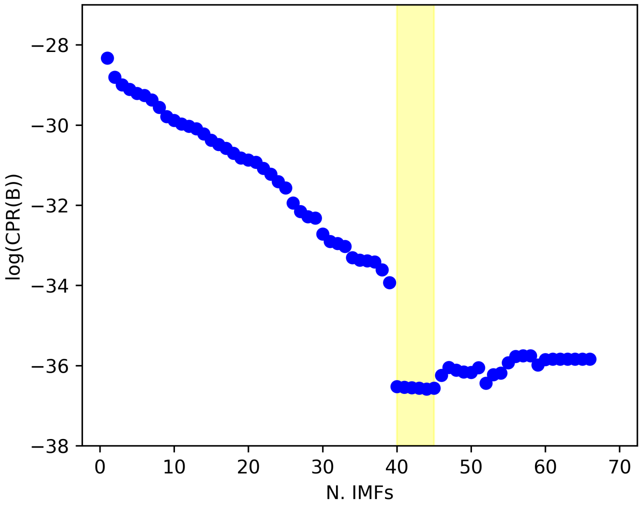

(b) with window function

FIGURE S12 Plot of CPR logarithm computed on the baseline as function of the number of IMFs excluded from its reconstruction, in the -125 ppm and -145 ppm region, for the experimental spectrum of Figure 5 with and without the application of the windowing function. The yellow region indicates the optimal number of IMFs that can be included in the signal reconstruction of Figure 5 (left and right panels respectively). The comparison of these plots highlights the higher quality of the decomposition obtained after the application of the periodic function compared to the original spectrum decomposition.

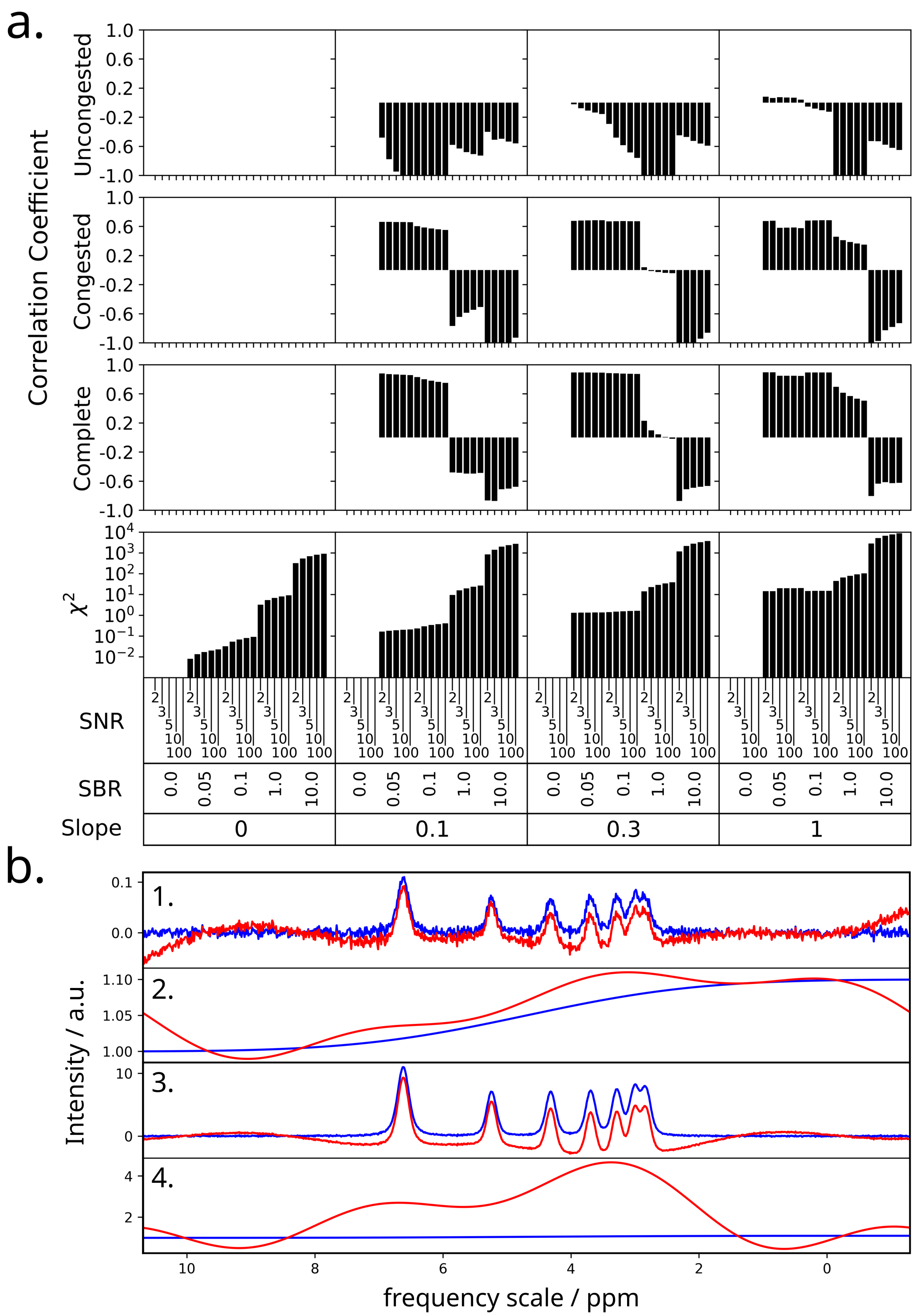

FIGURE S13 On panel a., FoM reproduced according to procedure described in section 2.3 for the spectral width of 12 ppm and asymettric baseline distortion. Panel b. reports the baseline and spectra reconstructed with FIF (red) and the original simulated signal (blue) for two parameters sets: SNR 10, SBR 0.1, Slope 0.1 in panels b.1 and b.2 and SNR 100, SBR 10, Slope 0.1 in panels b.3 and b.4.

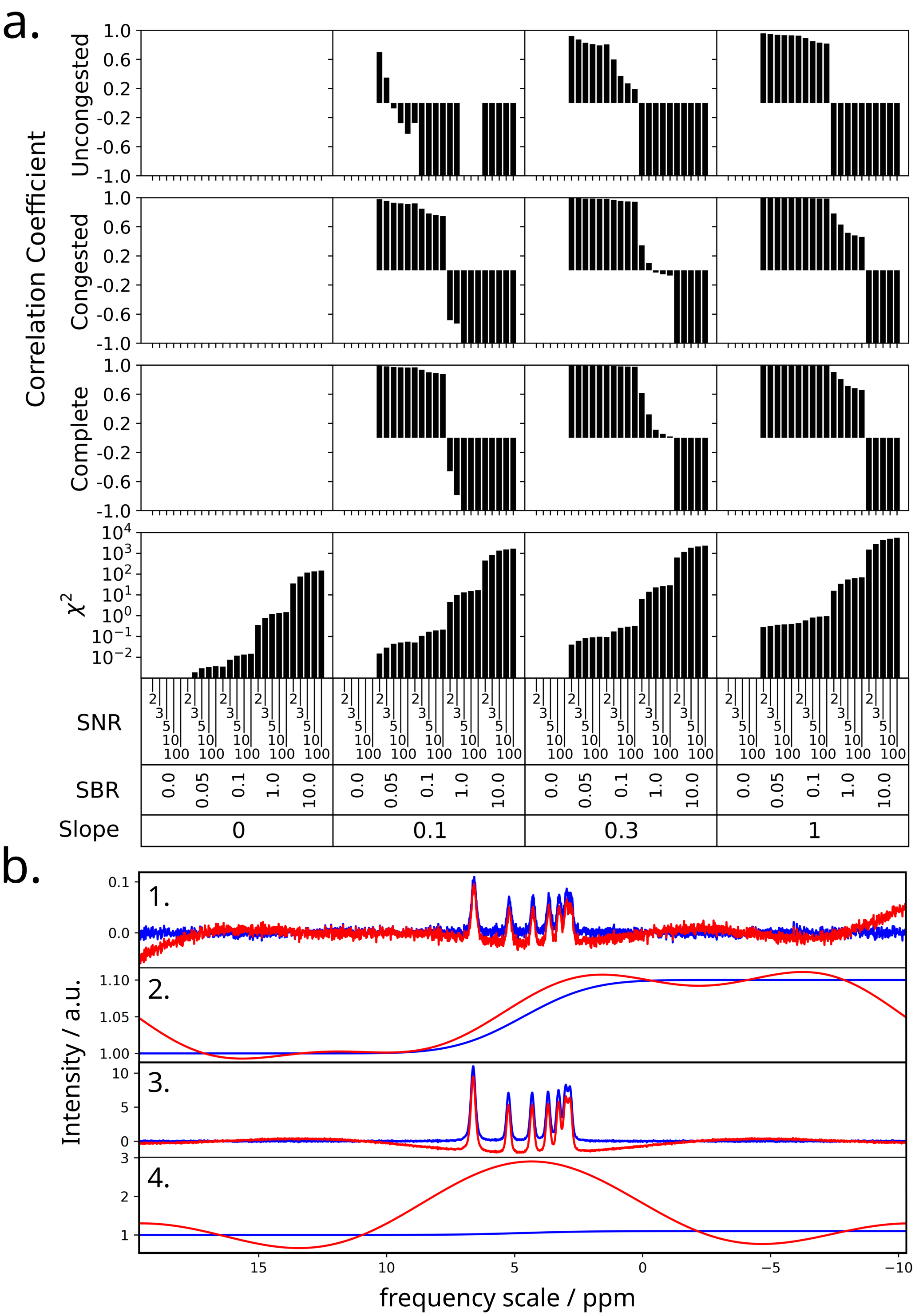

FIGURE S14 On panel a., FoM reproduced according to procedure described in section 2.3 for the spectral width of 30 ppm and asymetric baseline distortion. Panel b. reports the baseline and spectra reconstructed with FIF (red) and the original simulated signal (blue) for two parameters sets: SNR 10, SBR 0.1, Slope 0.1 in panels b.1 and b.2 and SNR 100, SBR 10, Slope 0.1 in panels b.3 and b.4.

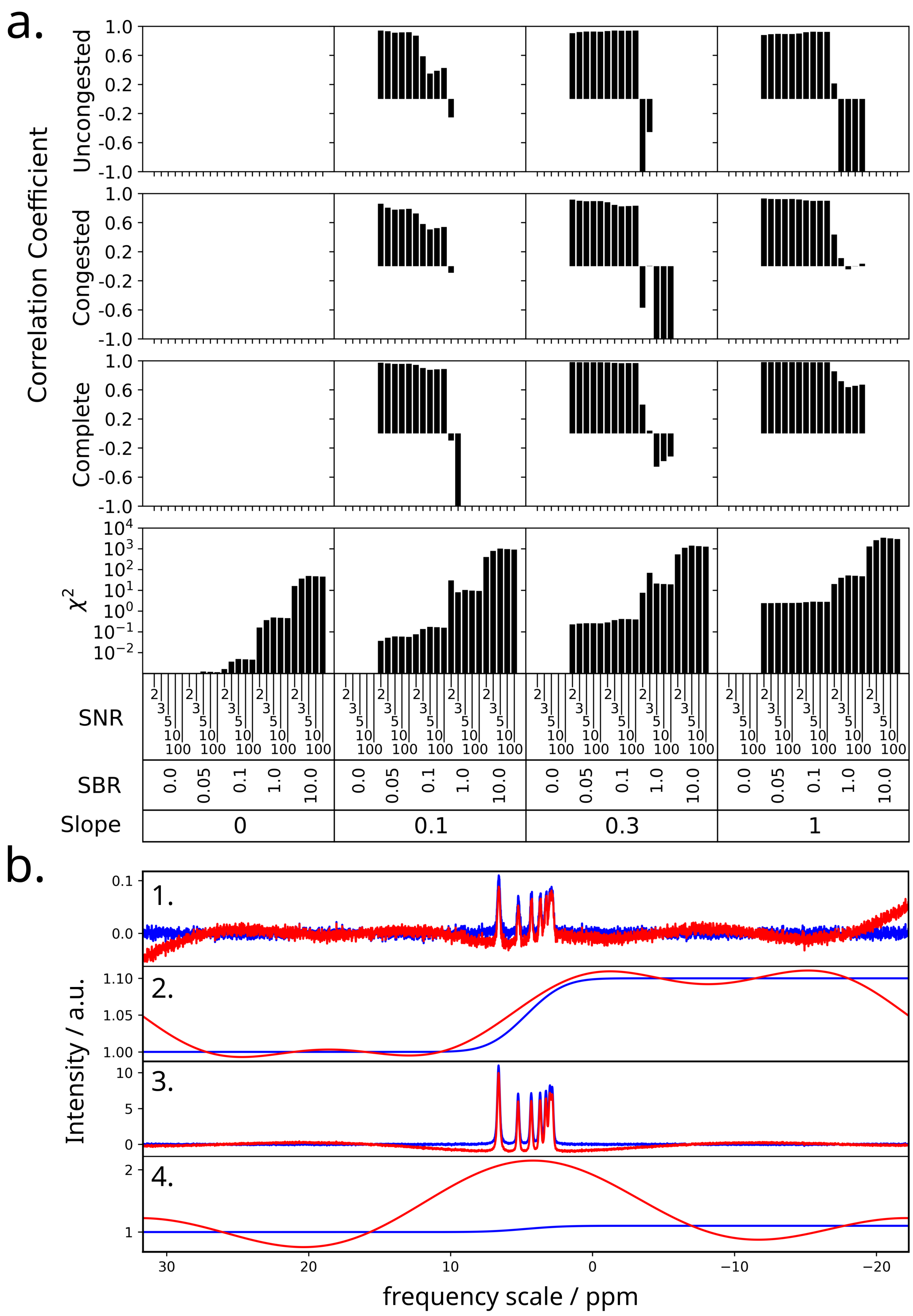

FIGURE S15 On panel a., FoM reproduced according to procedure described in section 2.3 for the spectral width of 54 ppm and asymetric baseline distortion. Panel b. reports the baseline and spectra reconstructed with FIF (red) and the original simulated signal (blue) for two parameters sets: SNR 10, SBR 0.1, Slope 0.1 in panels b.1 and b.2 and SNR 100, SBR 10, Slope 0.1 in panels b.3 and b.4.

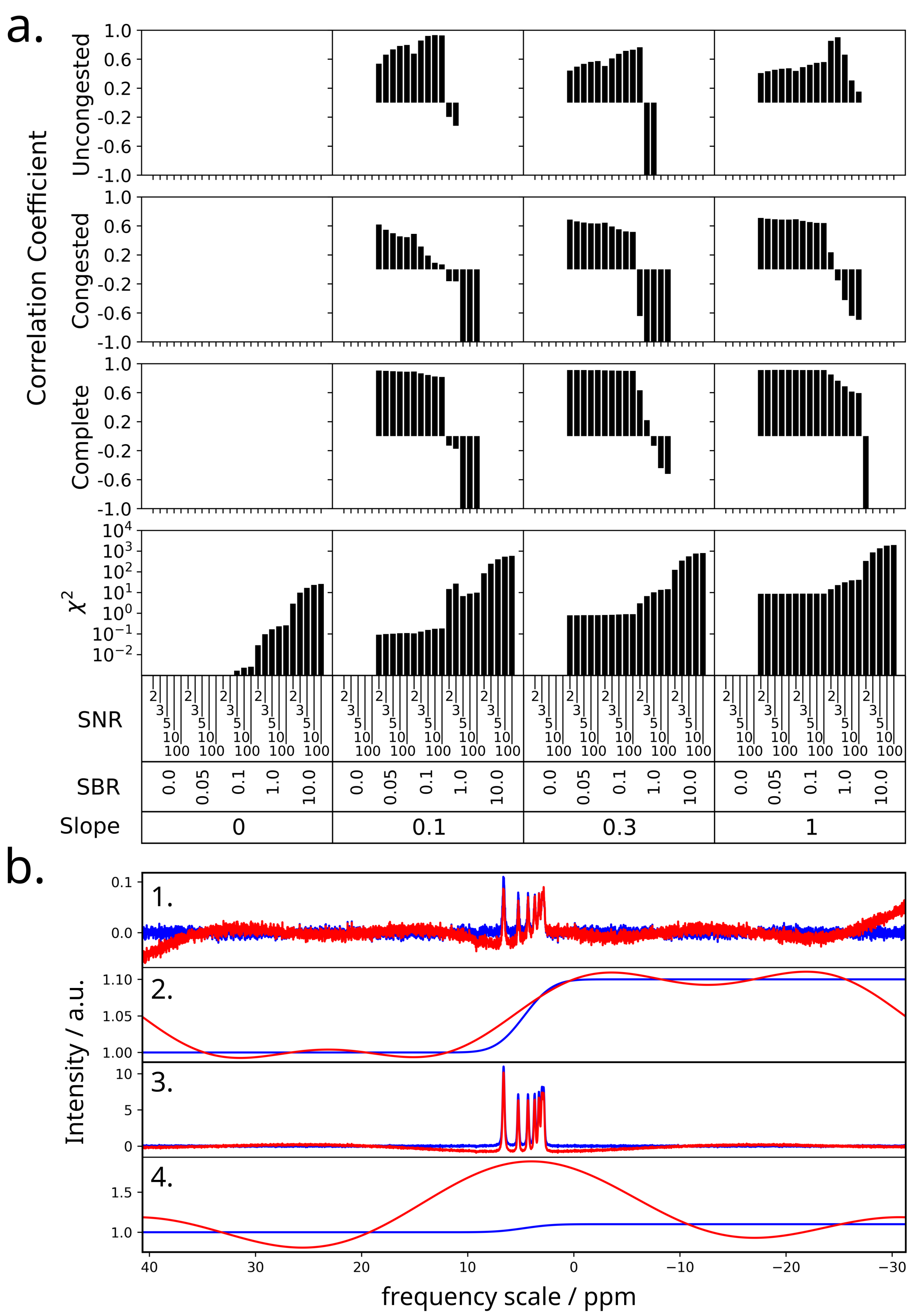

FIGURE S16 On panel a., FoM reproduced according to procedure described in section 2.3 for the spectral width of 72 ppm and asymetric baseline distortion. Panel b. reports the baseline and spectra reconstructed with FIF (red) and the original simulated signal (blue) for two parameters sets: SNR 10, SBR 0.1, Slope 0.1 in panels b.1 and b.2 and SNR 100, SBR 10, Slope 0.1 in panels b.3 and b.4.

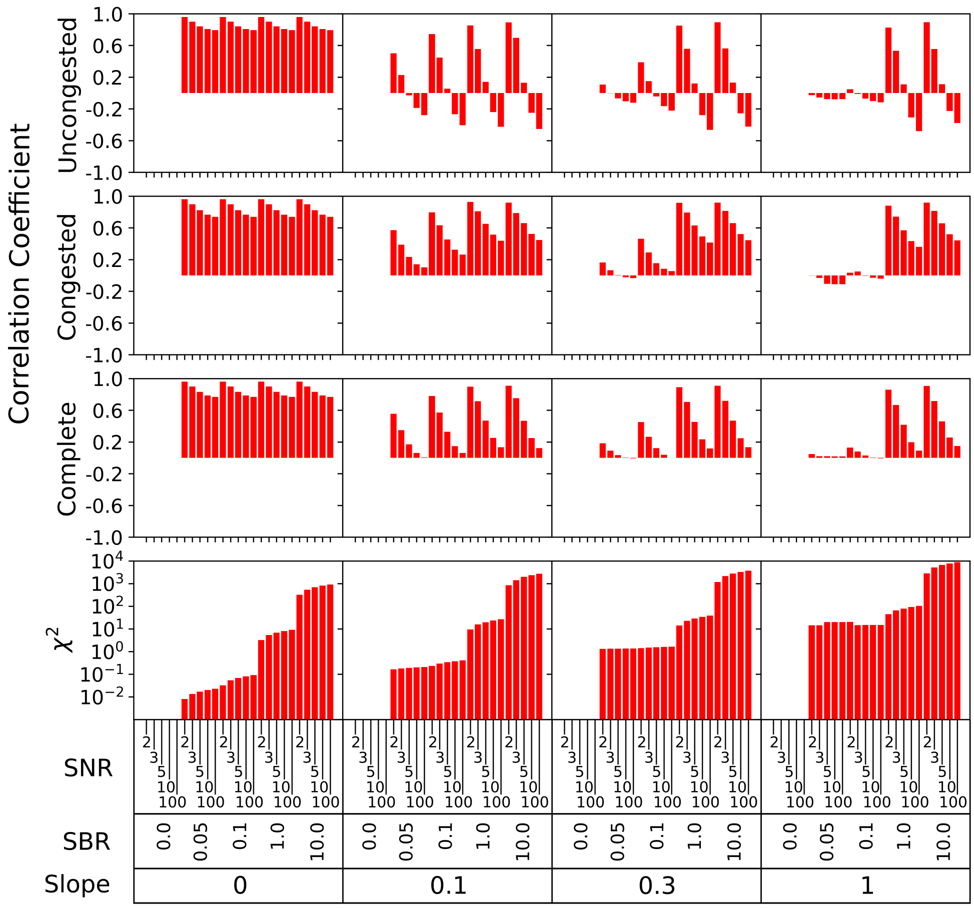

FIGURE S17 FoM reproduced according to procedure described in section 2.3 for the spectral width of 12 ppm and asymmetric baseline distortion. The correlation coefficients and χ^2 are computed with respect to the spectra reconstruction instead of the baseline.

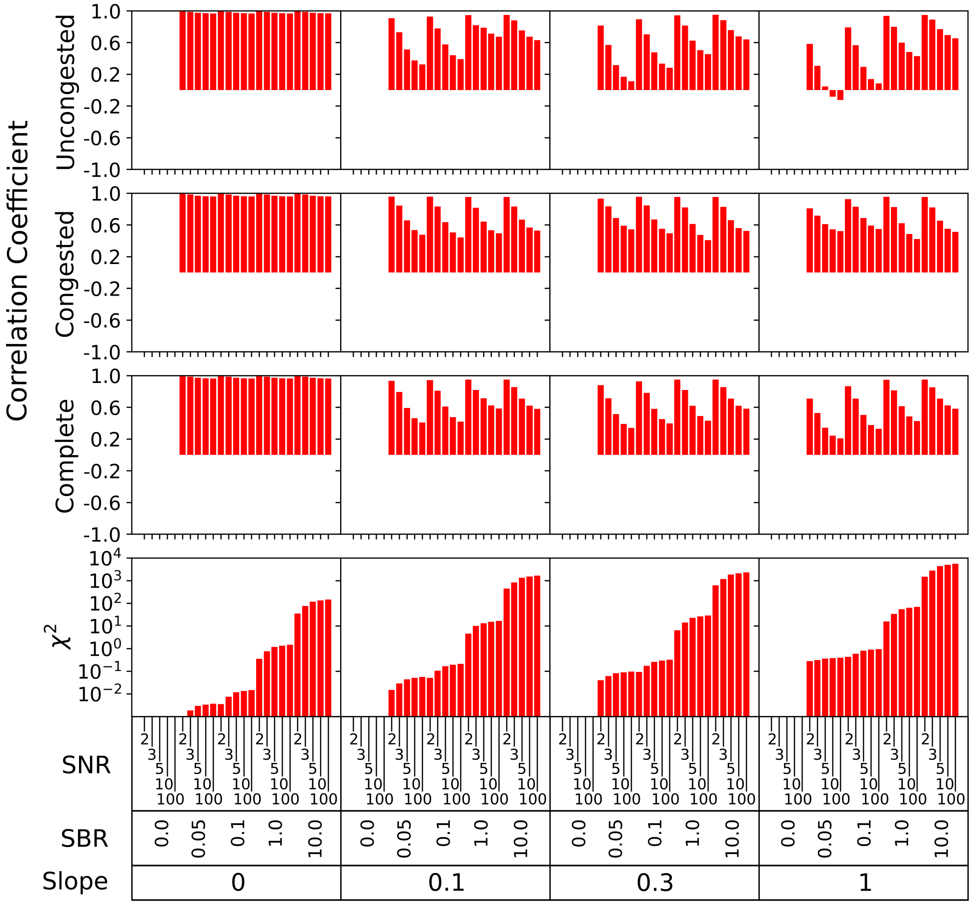

FIGURE S18 FoM reproduced according to procedure described in section 2.3 for the spectral width of 30 ppm and asymmetric baseline distortion. The correlation coefficients and χ^2 are computed with respect to the spectra reconstruction instead of the baseline.

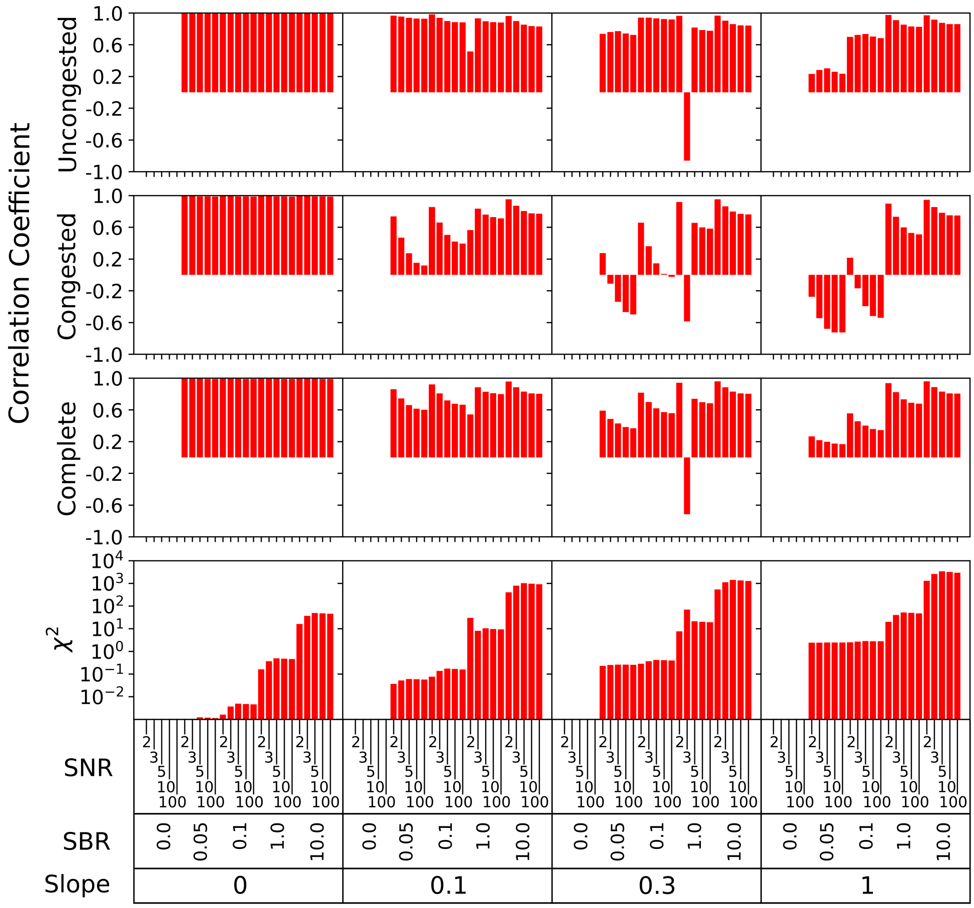

FIGURE S19 FoM reproduced according to procedure described in section 2.3 for the spectral width of 54 ppm and asymmetric baseline distortion. The correlation coefficients and χ^2 are computed with respect to the spectra reconstruction instead of the baseline.

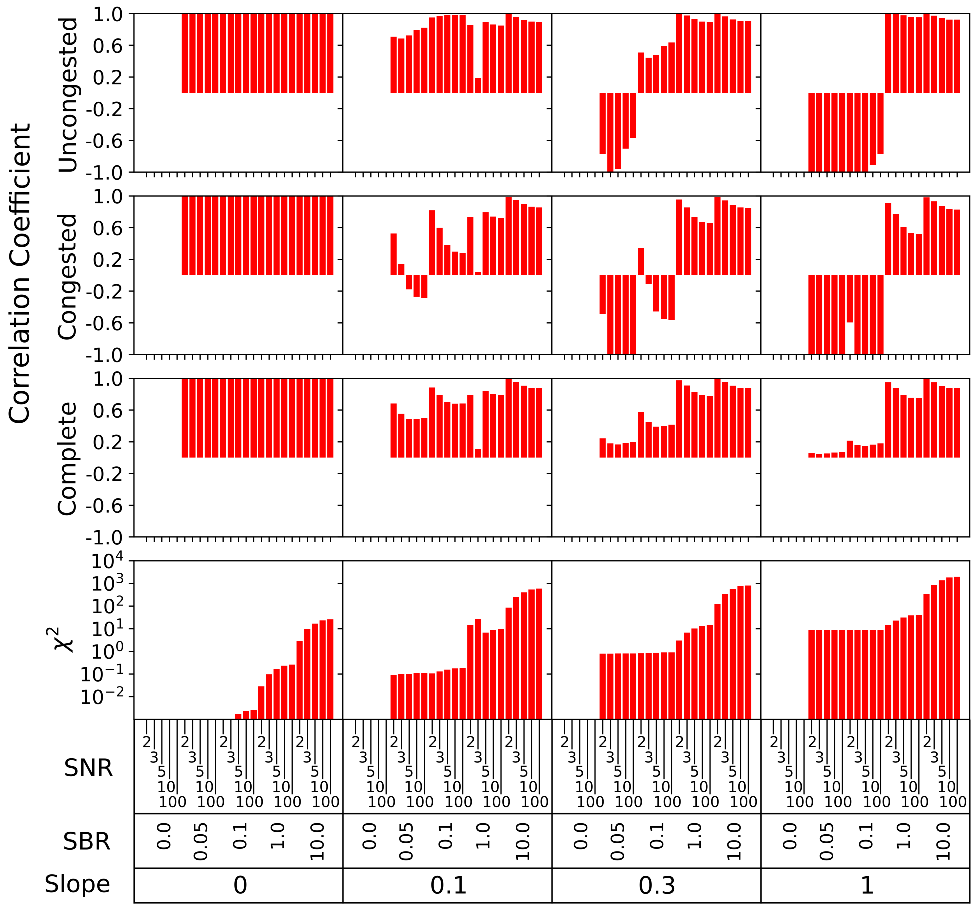

FIGURE S20 FoM reproduced according to procedure described in section 2.3 for the spectral width of 72 ppm and asymmetric baseline distortion. The correlation coefficients and χ^2 are computed with respect to the spectra reconstruction instead of the baseline.

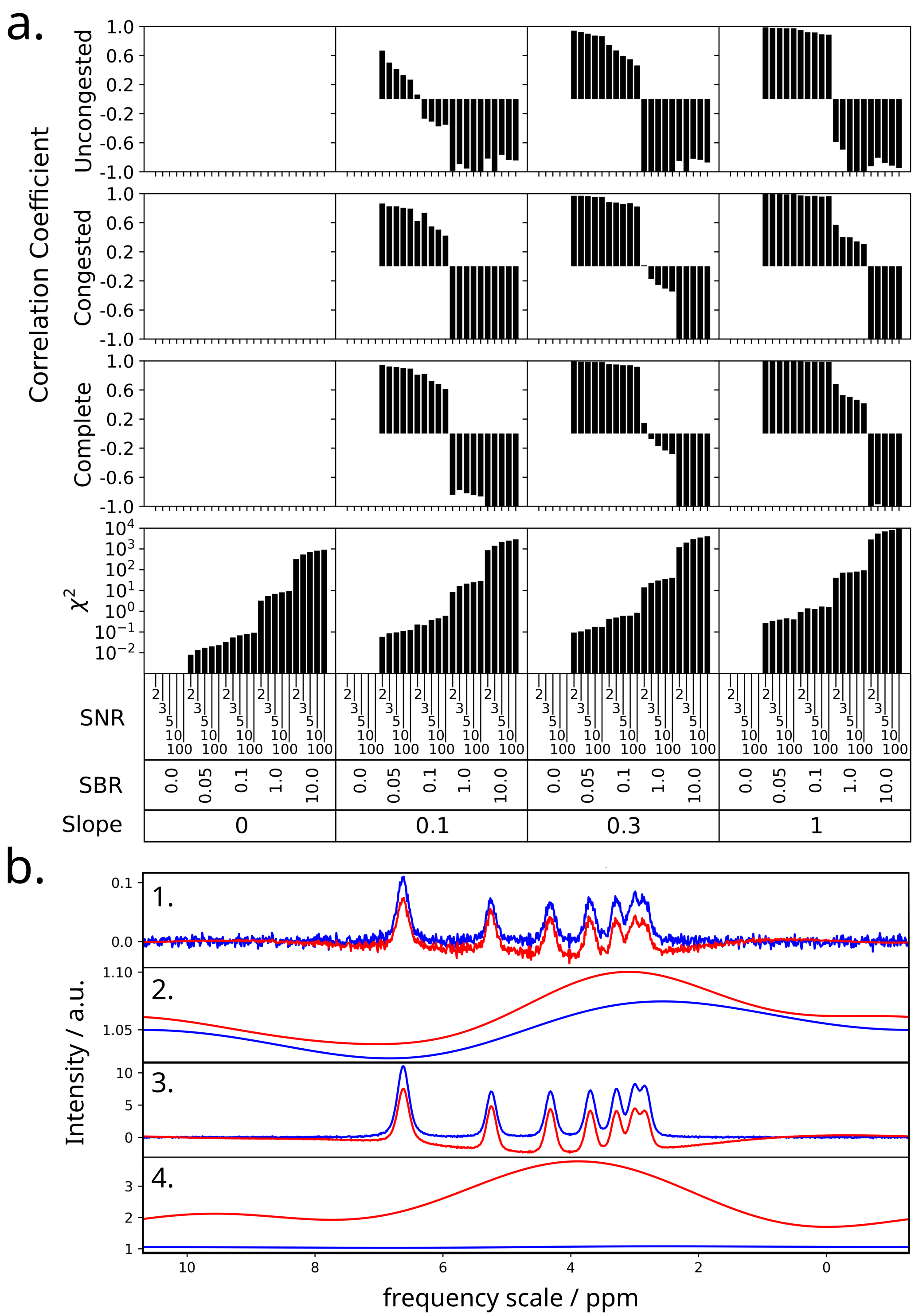

FIGURE S21 On panel a., FoM reproduced according to procedure described in section 2.3 for the spectral width of 12 ppm and asymettric baseline distortion and windowing function. Panel b. reports the baseline and spectra reconstructed with FIF (red) and the original simulated signal (blue) for two parameters sets: SNR 10, SBR 0.1, Slope 0.1 in panels b.1 and b.2 and SNR 100, SBR 10, Slope 0.1 in panels b.3 and b.4.

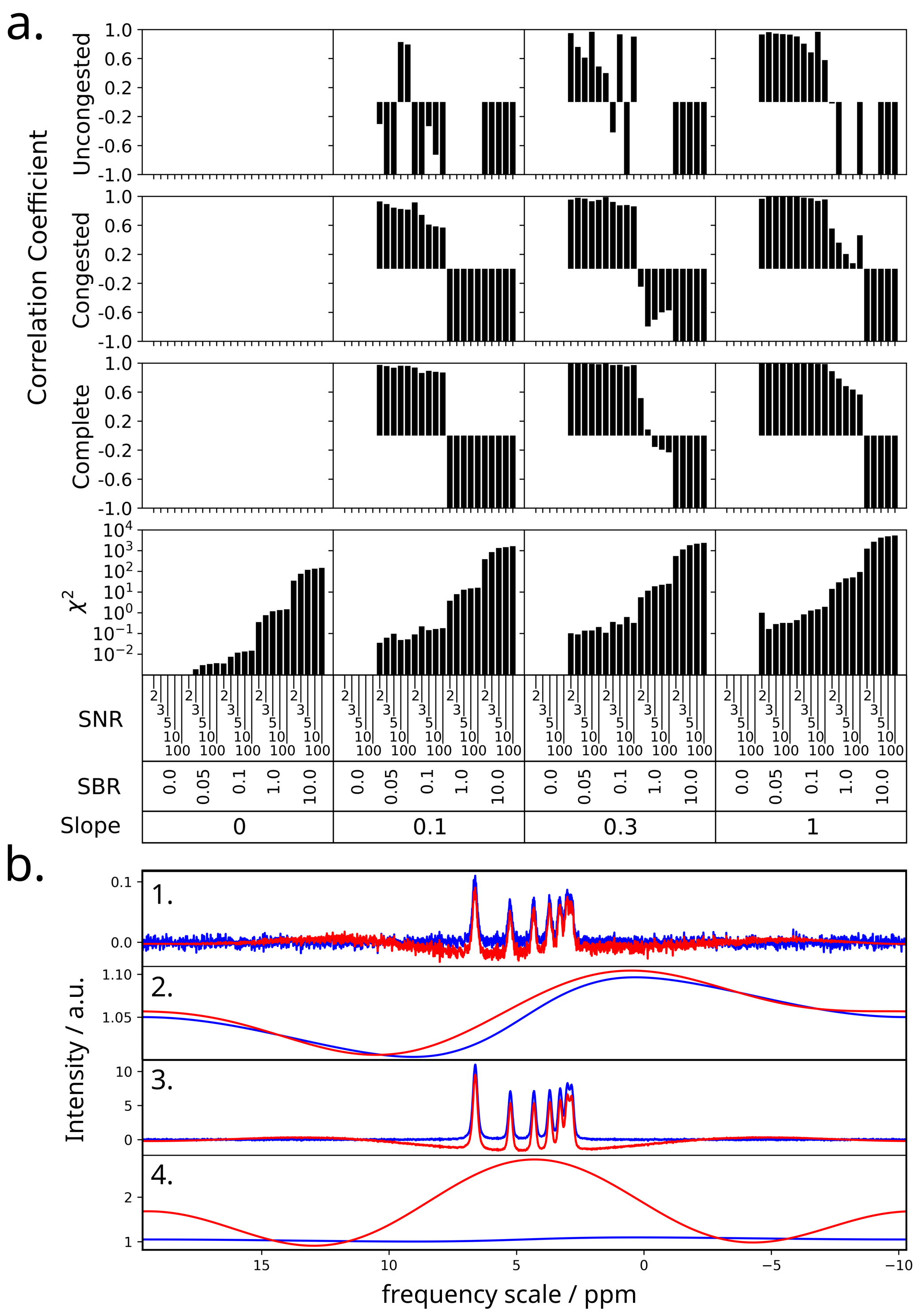

FIGURE S22 On panel a., FoM reproduced according to procedure described in section 2.3 for the spectral width of 30 ppm and asymettric baseline distortion and windowing function. Panel b. reports the baseline and spectra reconstructed with FIF (red) and the original simulated signal (blue) for two parameters sets: SNR 10, SBR 0.1, Slope 0.1 in panels b.1 and b.2 and SNR 100, SBR 10, Slope 0.1 in panels b.3 and b.4.

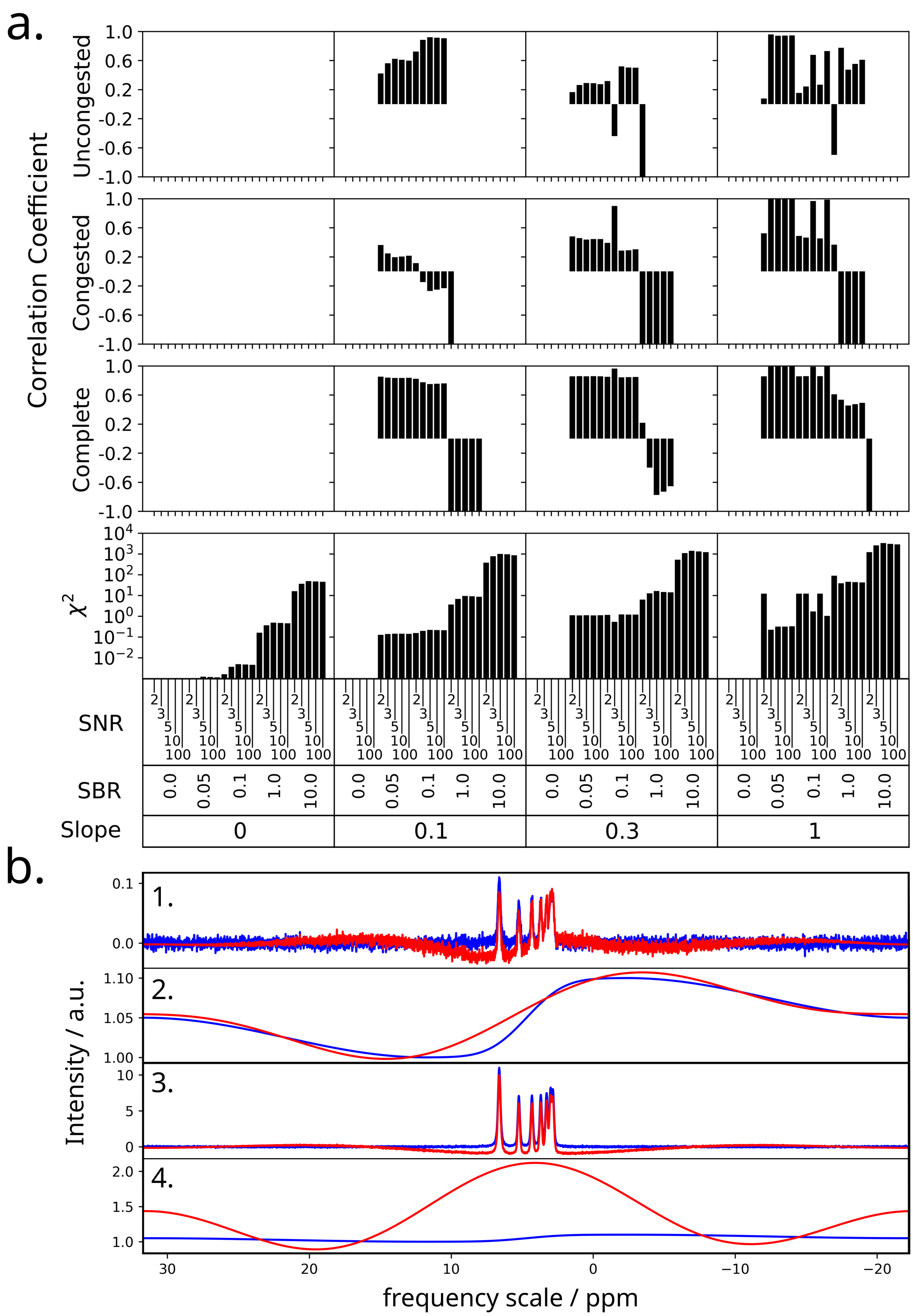

FIGURE S23 On panel a., FoM reproduced according to procedure described in section 2.3 for the spectral width of 54 ppm and asymettric baseline distortion and windowing function. Panel b. reports the baseline and spectra reconstructed with FIF (red) and the original simulated signal (blue) for two parameters sets: SNR 10, SBR 0.1, Slope 0.1 in panels b.1 and b.2 and SNR 100, SBR 10, Slope 0.1 in panels b.3 and b.4.

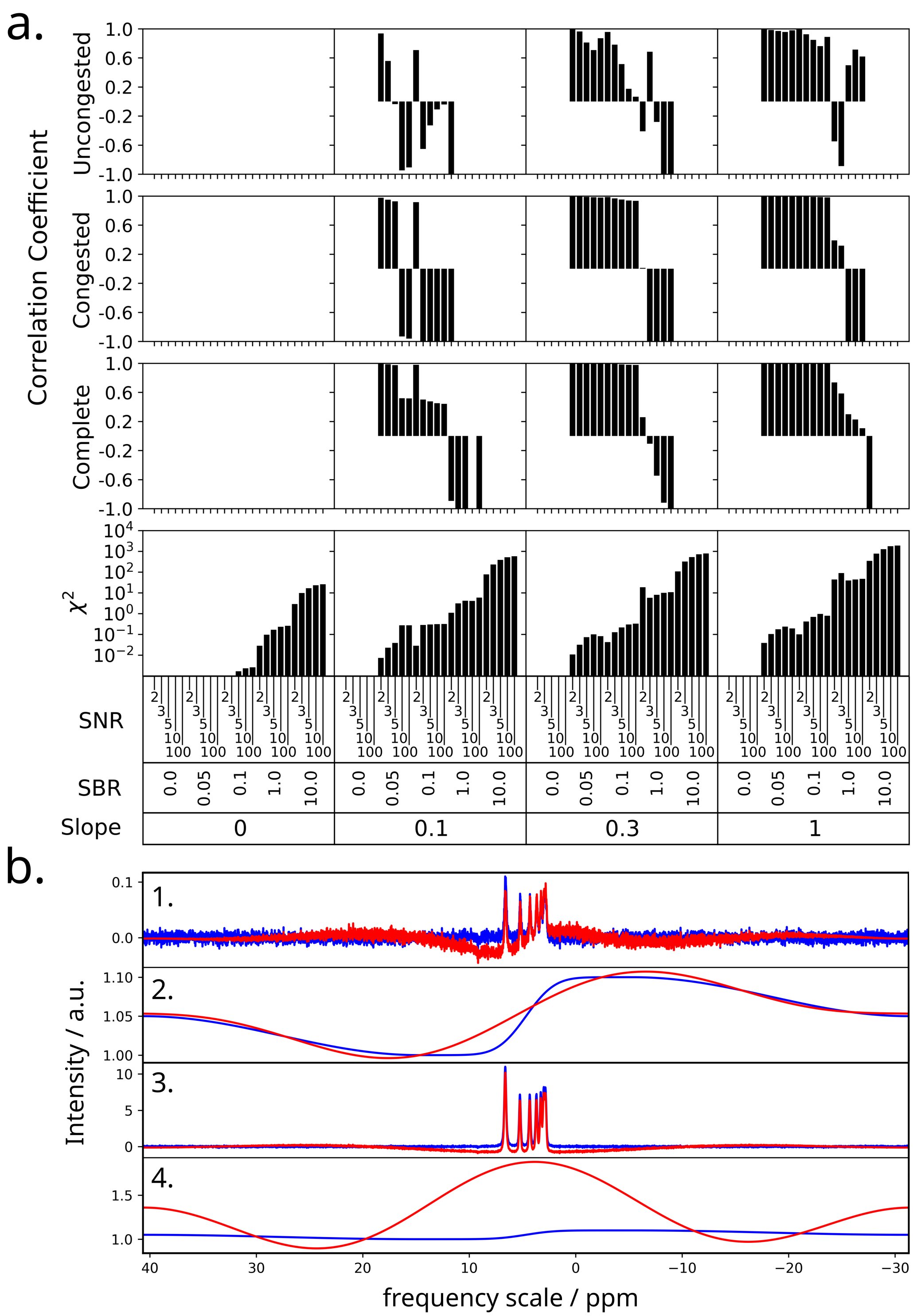

FIGURE S24 On panel a., FoM reproduced according to procedure described in section 2.3 for the spectral width of 72 ppm and asymettric baseline distortion and windowing function. Panel b. reports the baseline and spectra reconstructed with FIF (red) and the original simulated signal (blue) for two parameters sets: SNR 10, SBR 0.1, Slope 0.1 in panels b.1 and b.2 and SNR 100, SBR 10, Slope 0.1 in panels b.3 and b.4.

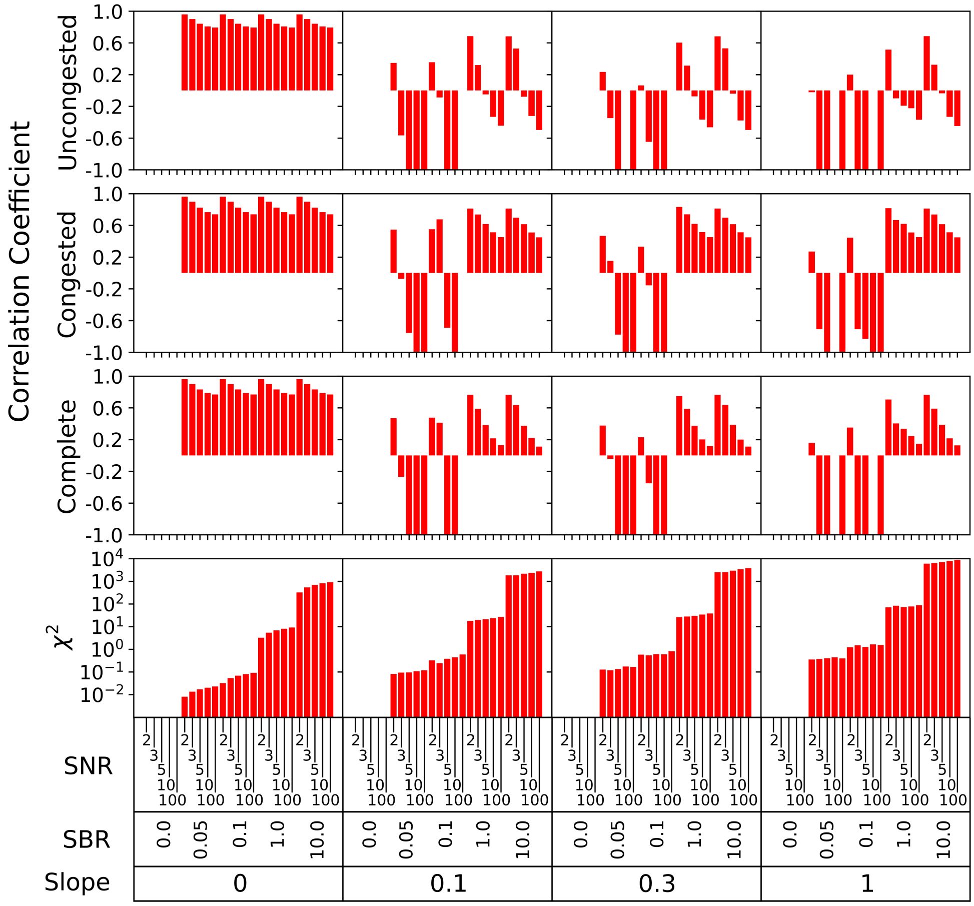

FIGURE S25 FoM reproduced according to procedure described in section 2.3 for the spectral width of 12 ppm and asymmetric baseline distortion and application of the widwing function. The correlation coefficients and χ^2 are computed with respect to the spectra reconstruction instead of the baseline.

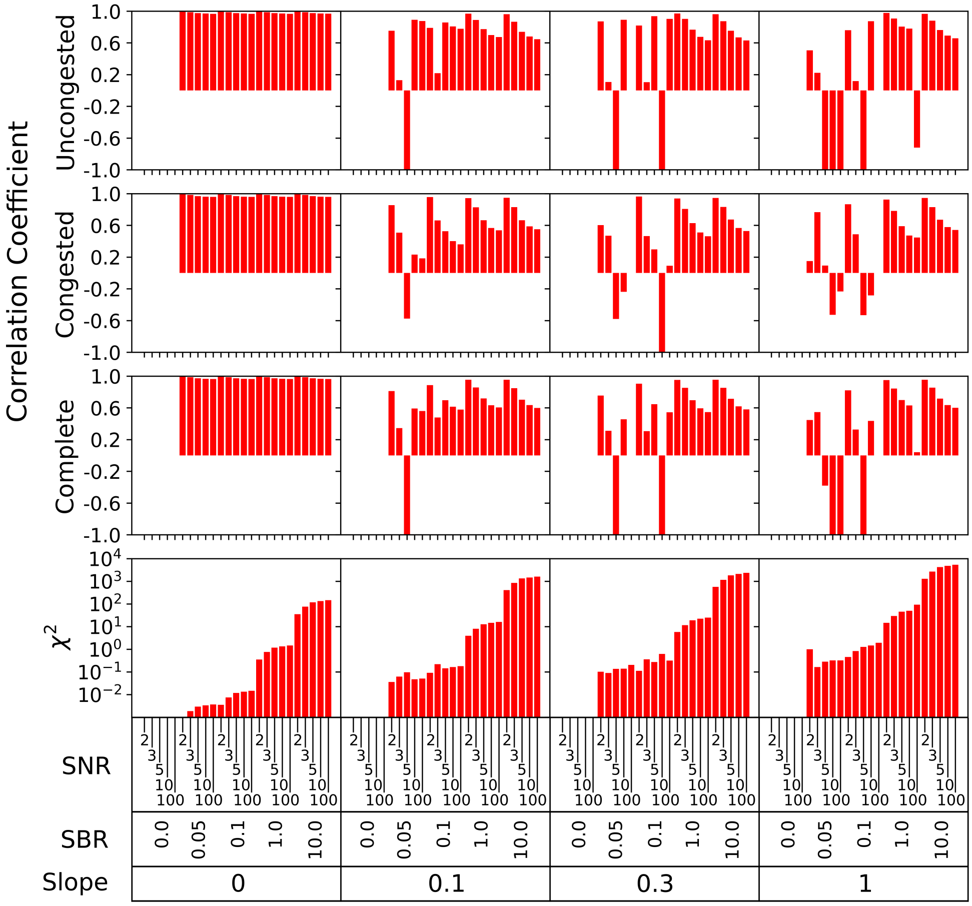

FIGURE S26 FoM reproduced according to procedure described in section 2.3 for the spectral width of 30 ppm and asymmetric baseline distortion and application of the widwing function. The correlation coefficients and χ^2 are computed with respect to the spectra reconstruction instead of the baseline.

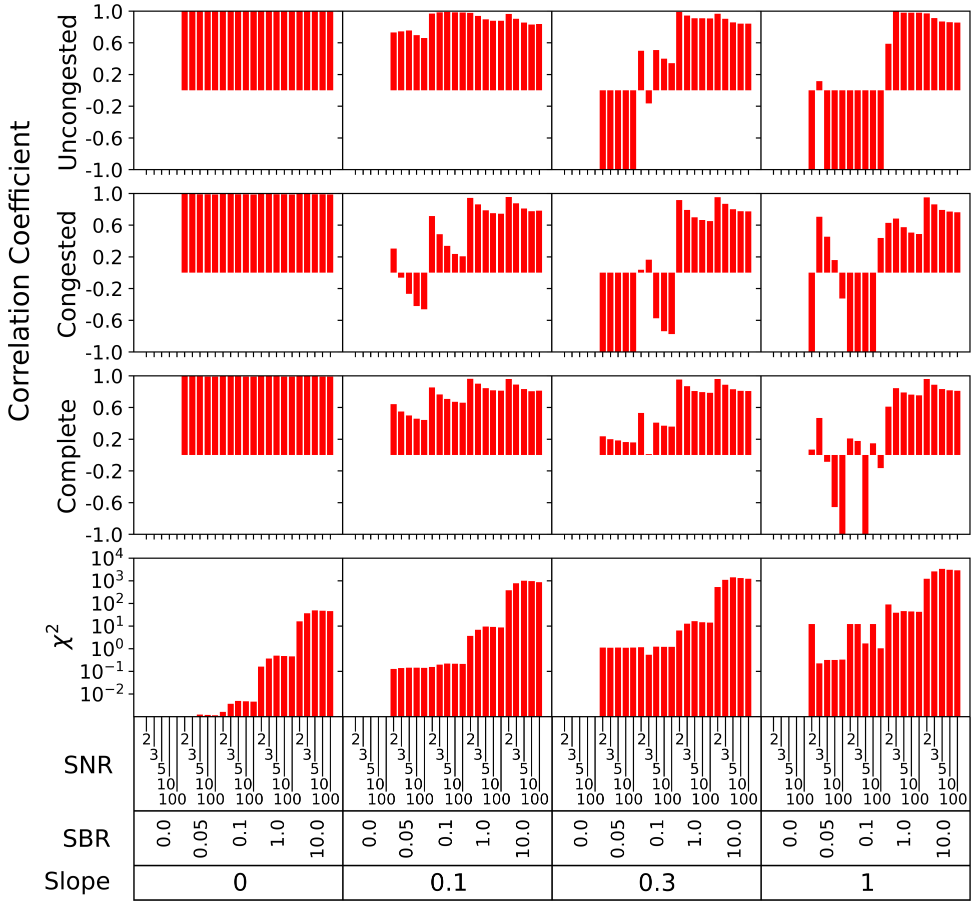

FIGURE S27 FoM reproduced according to procedure described in section 2.3 for the spectral width of 54 ppm and asymmetric baseline distortion and application of the widwing function. The correlation coefficients and χ^2 are computed with respect to the spectra reconstruction instead of the baseline.

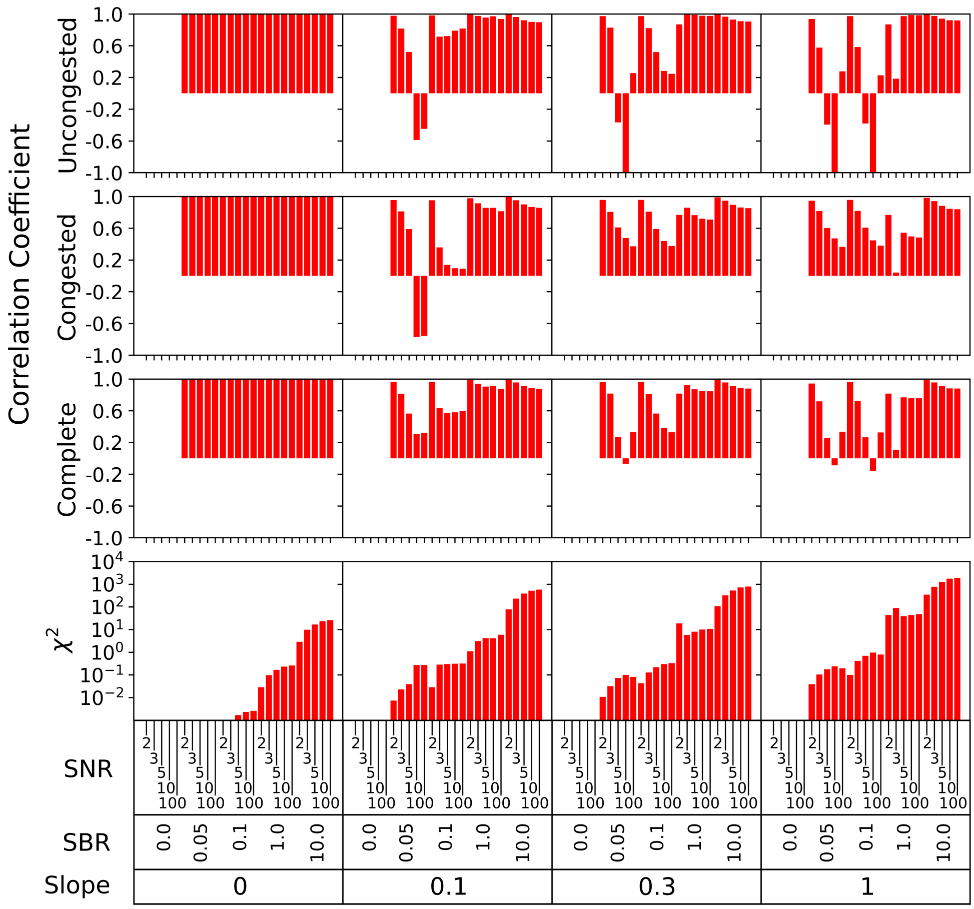

FIGURE S28 FoM reproduced according to procedure described in section 2.3 for the spectral width of 72 ppm and asymmetric baseline distortion and application of the widwing function. The correlation coefficients and χ^2 are computed with respect to the spectra reconstruction instead of the baseline.

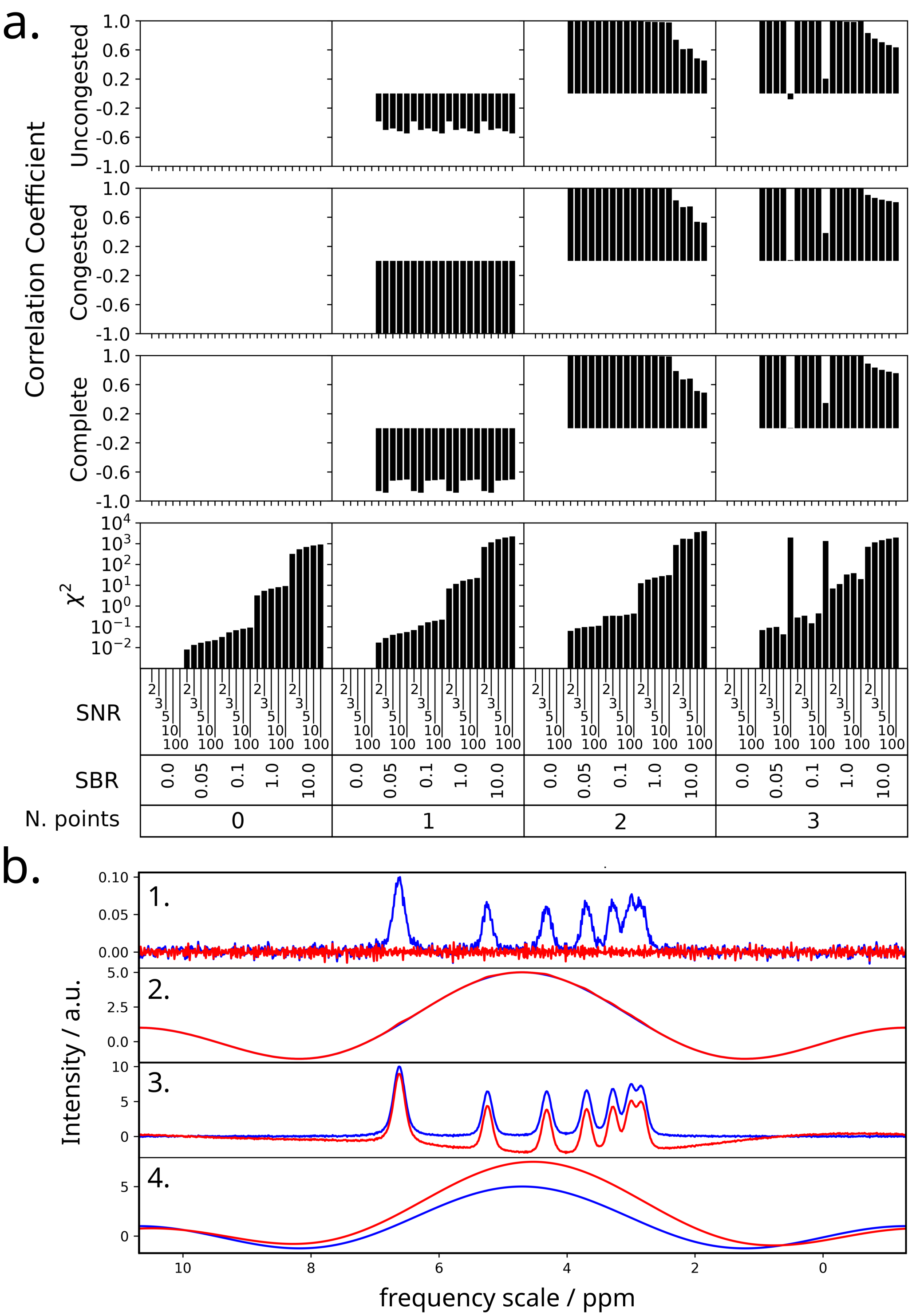

FIGURE S29 On panel a., FoM reproduced according to procedure described in section 2.3 for the spectral width of 12 ppm and baseline rolling. Panel b. reports the baseline and spectra reconstructed with FIF (red) and the original simulated signal (blue) for two parameters sets: SNR 10, SBR 0.1, N. points 3 in panels b.1 and b.2 and SNR 100, SBR 10, N. points 3 in panels b.3 and b.4.

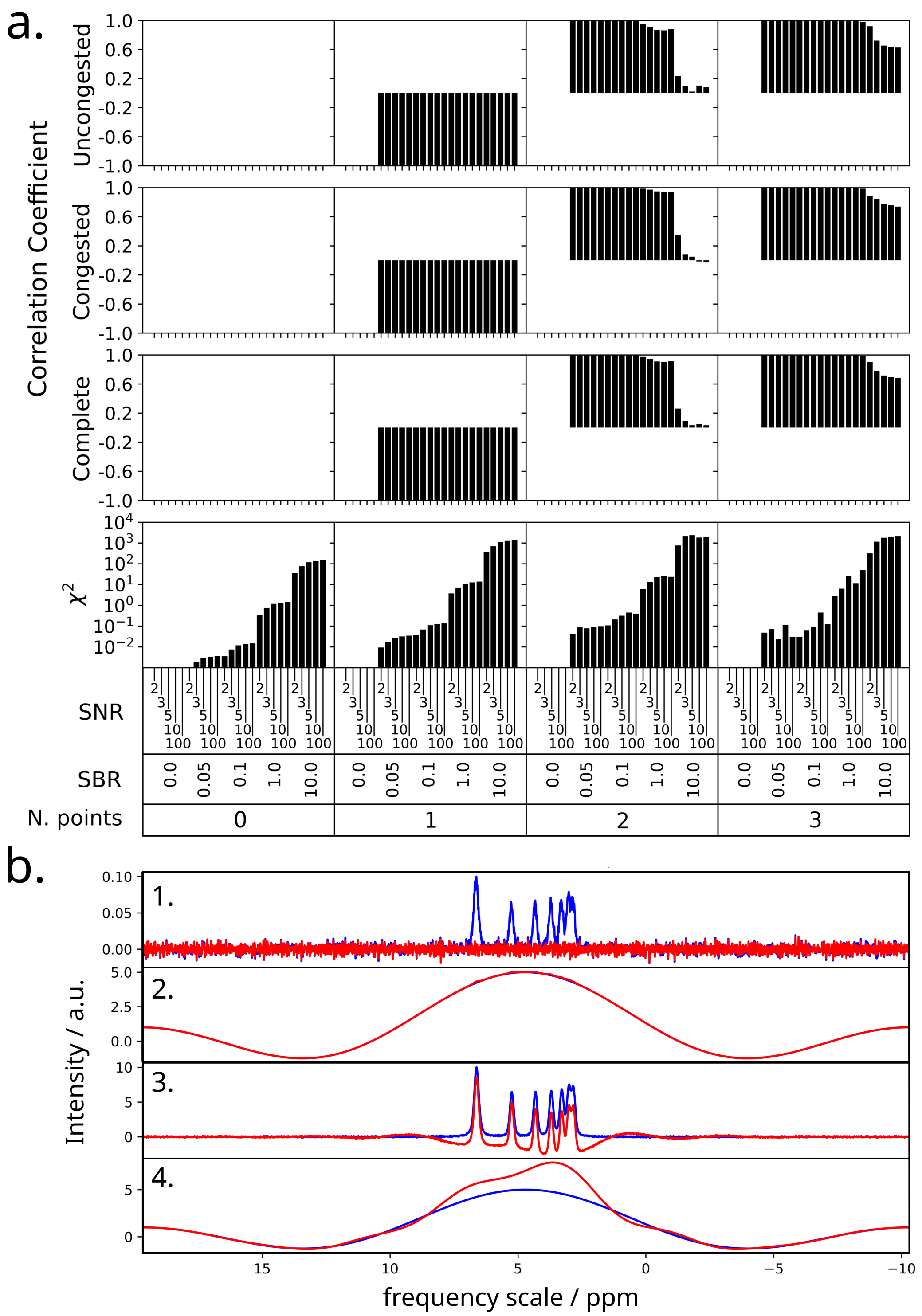

FIGURE S30 On panel a., FoM reproduced according to procedure described in section 2.3 for the spectral width of 30 ppm and baseline rolling. Panel b. reports the baseline and spectra reconstructed with FIF (red) and the original simulated signal (blue) for two parameters sets: SNR 10, SBR 0.1, N. points 3 in panels b.1 and b.2 and SNR 100, SBR 10, N. points 3 in panels b.3 and b.4.

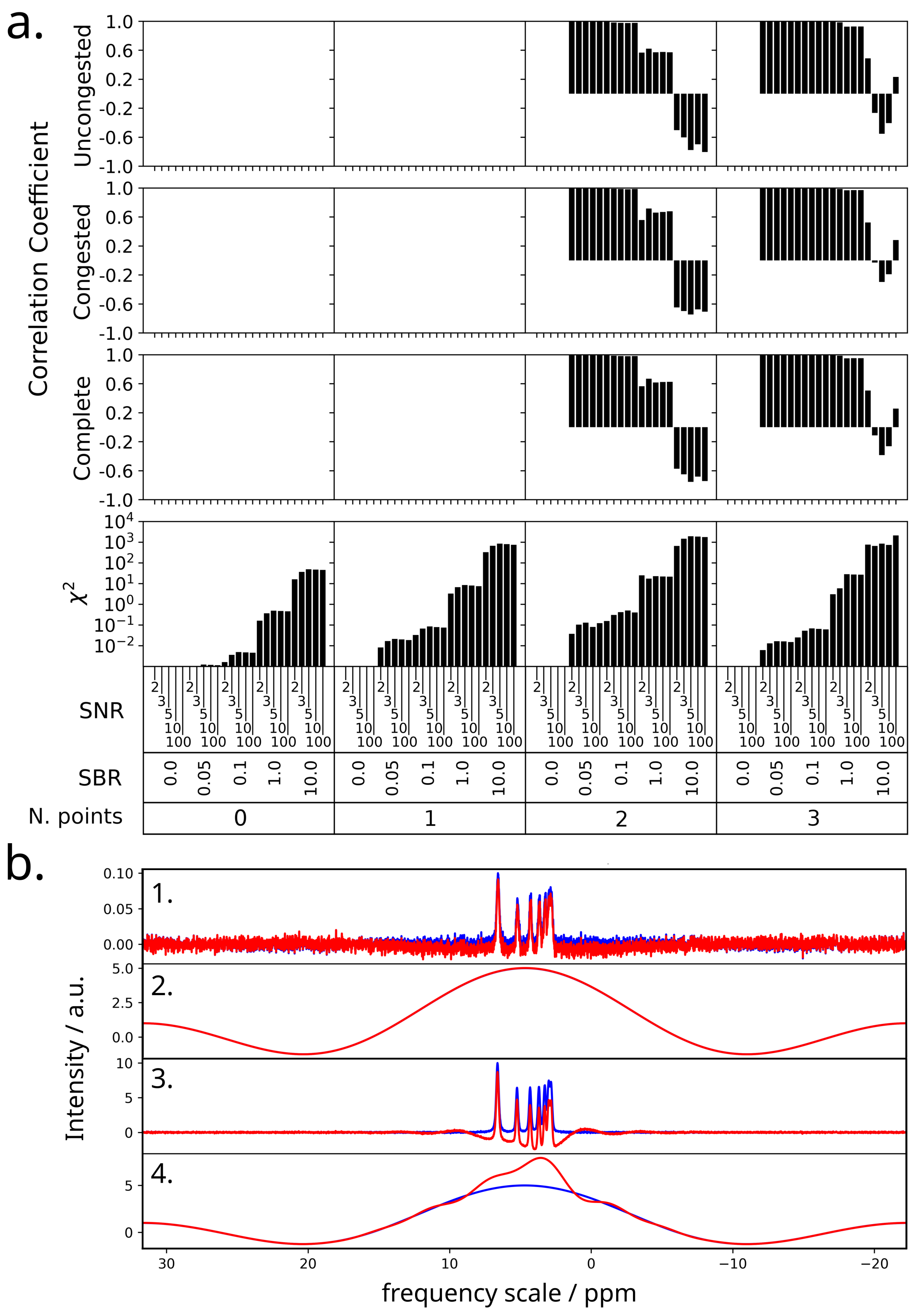

FIGURE S31 On panel a., FoM reproduced according to procedure described in section 2.3 for the spectral width of 54 ppm and baseline rolling. Panel b. reports the baseline and spectra reconstructed with FIF (red) and the original simulated signal (blue) for two parameters sets: SNR 10, SBR 0.1, N. points 3 in panels b.1 and b.2 and SNR 100, SBR 10, N. points 3 in panels b.3 and b.4.

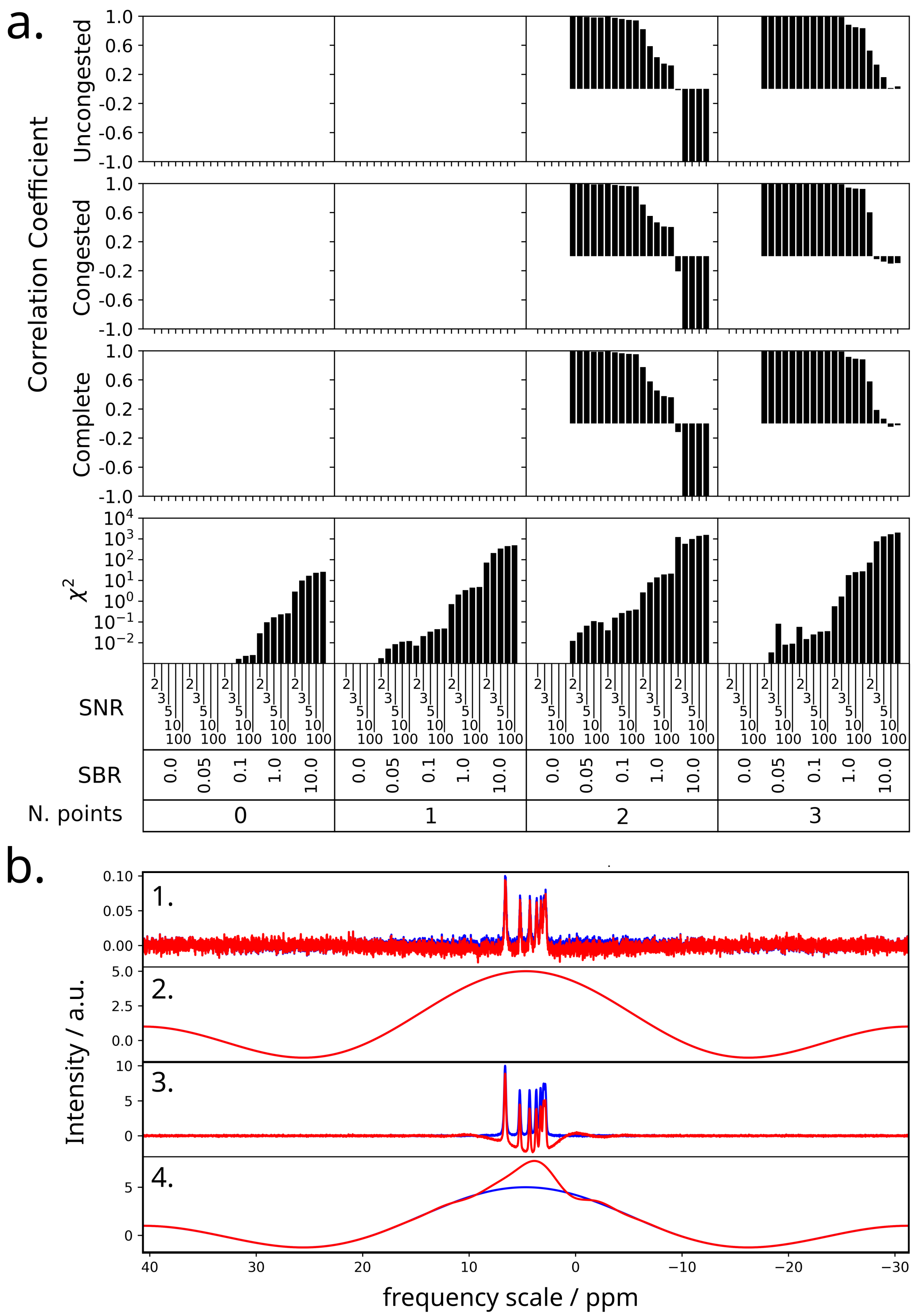

FIGURE S32 On panel a., FoM reproduced according to procedure described in section 2.3 for the spectral width of 72 ppm and baseline rolling. Panel b. reports the baseline and spectra reconstructed with FIF (red) and the original simulated signal (blue) for two parameters sets: SNR 10, SBR 0.1, N. points 3 in panels b.1 and b.2 and SNR 100, SBR 10, N. points 3 in panels b.3 and b.4.

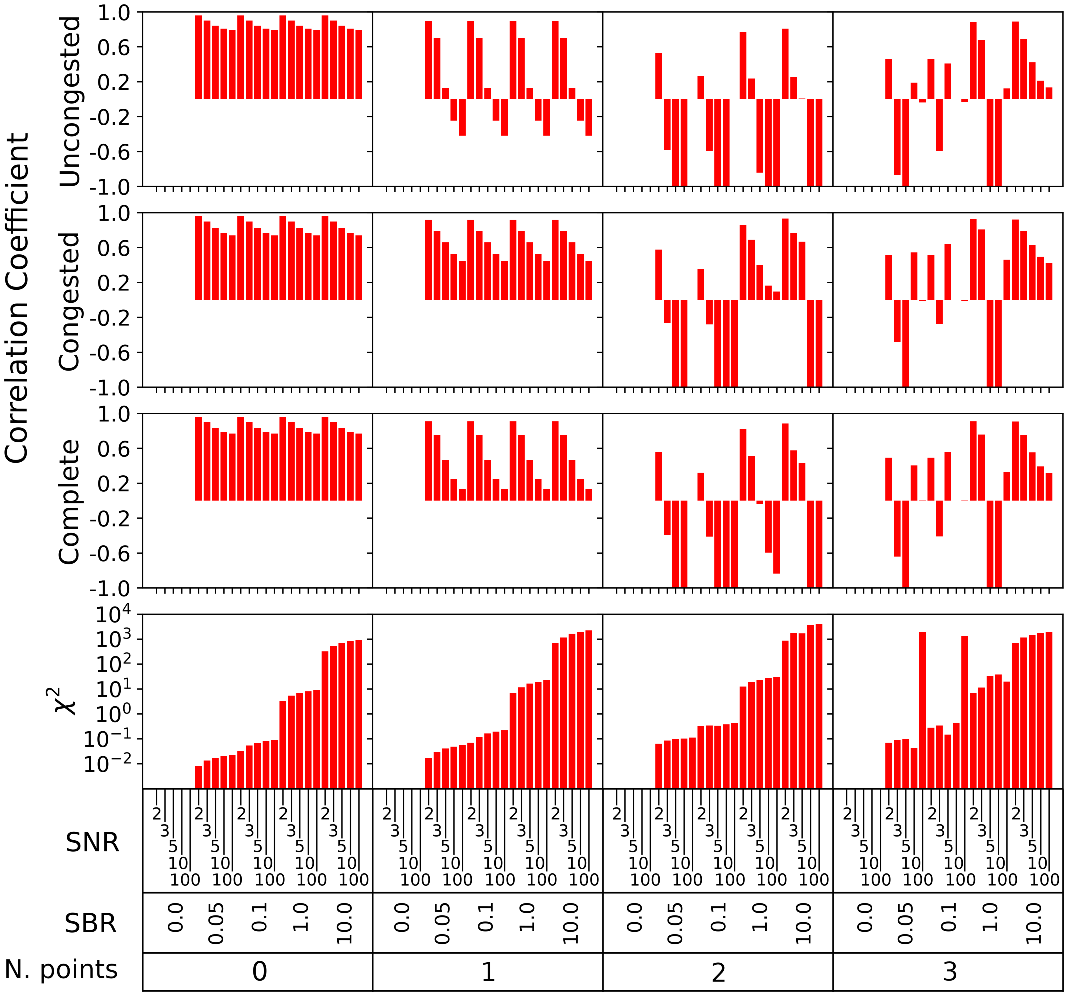

FIGURE S33 FoM reproduced according to procedure described in section 2.3 for the spectral width of 12 ppm and baseline rolling. The correlation coefficients and χ^2 are computed with respect to the spectra reconstruction instead of the baseline.

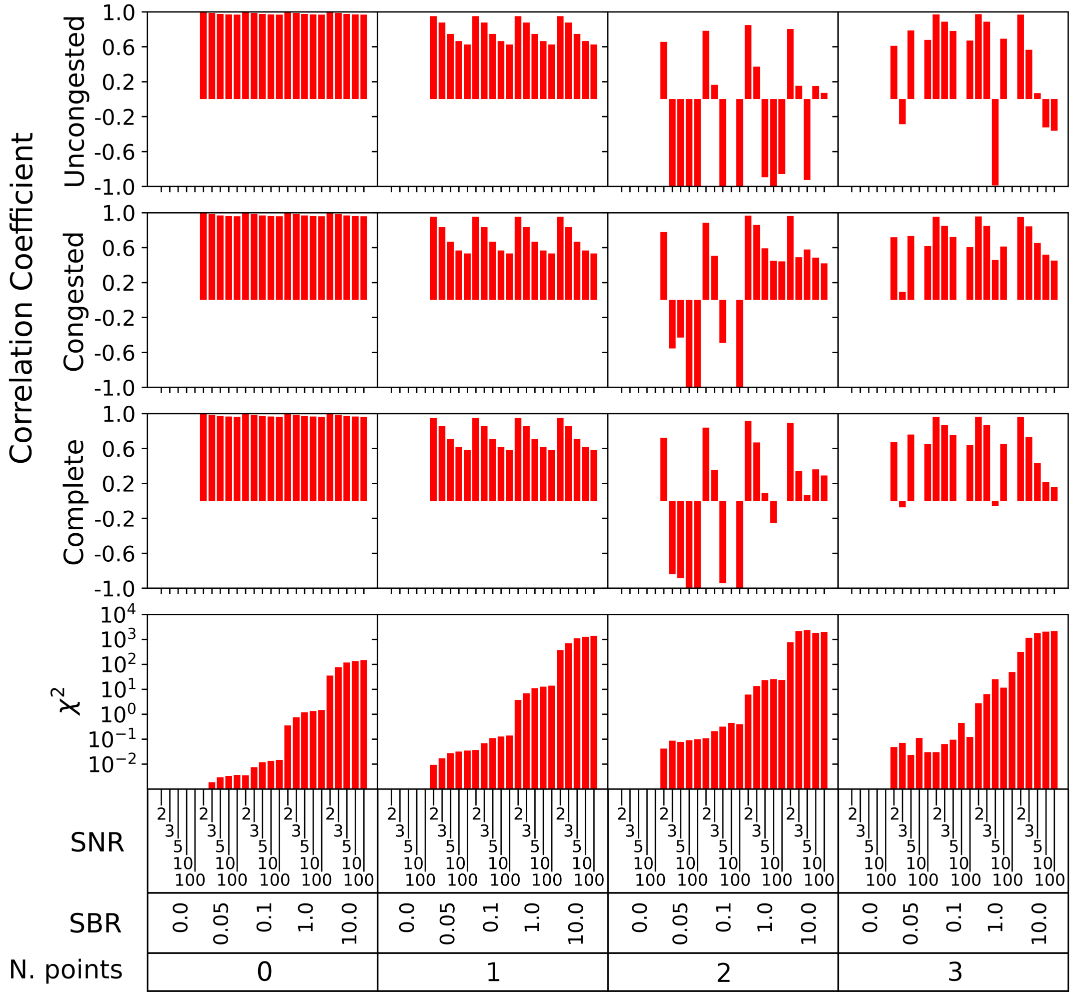

FIGURE S34 FoM reproduced according to procedure described in section 2.3 for the spectral width of 30 ppm and baseline rolling. The correlation coefficients and χ^2 are computed with respect to the spectra reconstruction instead of the baseline.

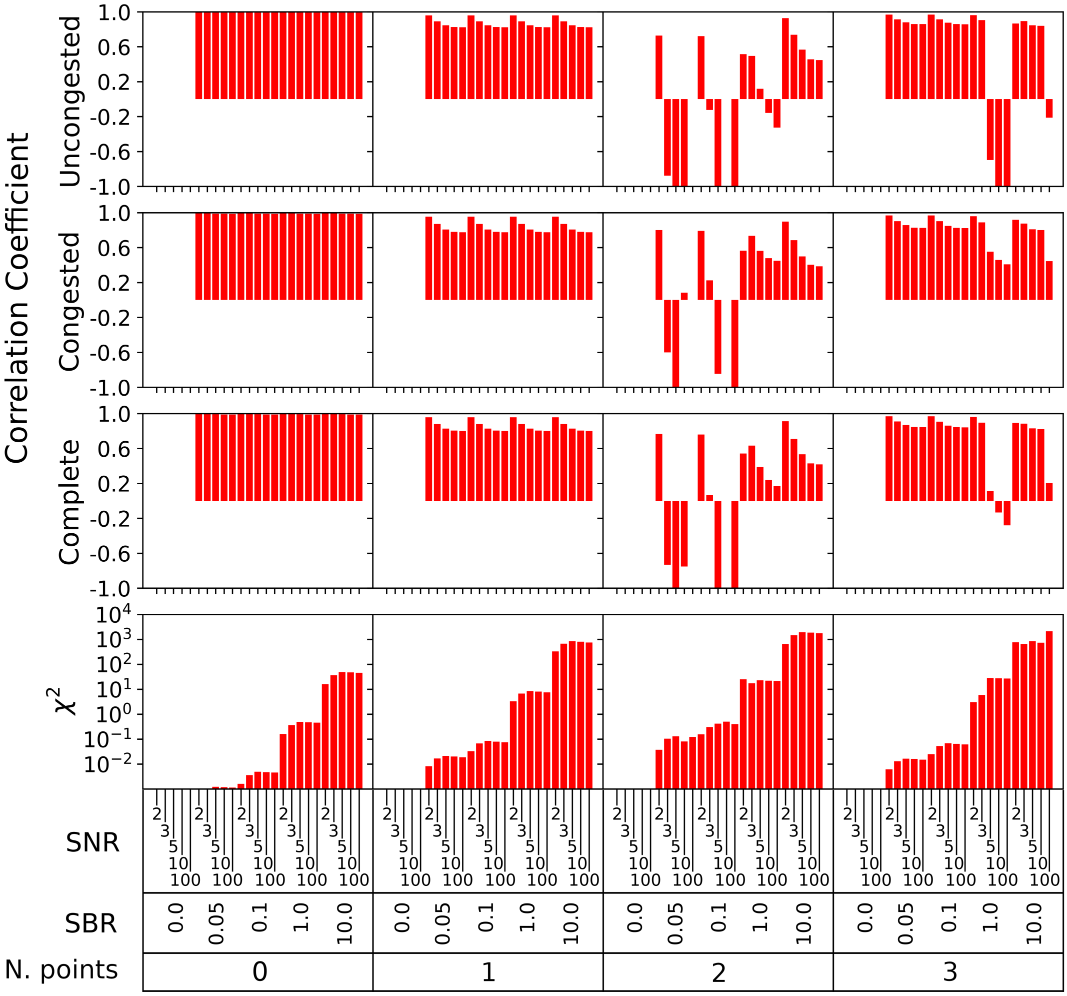

FIGURE S35 FoM reproduced according to procedure described in section 2.3 for the spectral width of 54 ppm and baseline rolling. The correlation coefficients and χ^2 are computed with respect to the spectra reconstruction instead of the baseline.

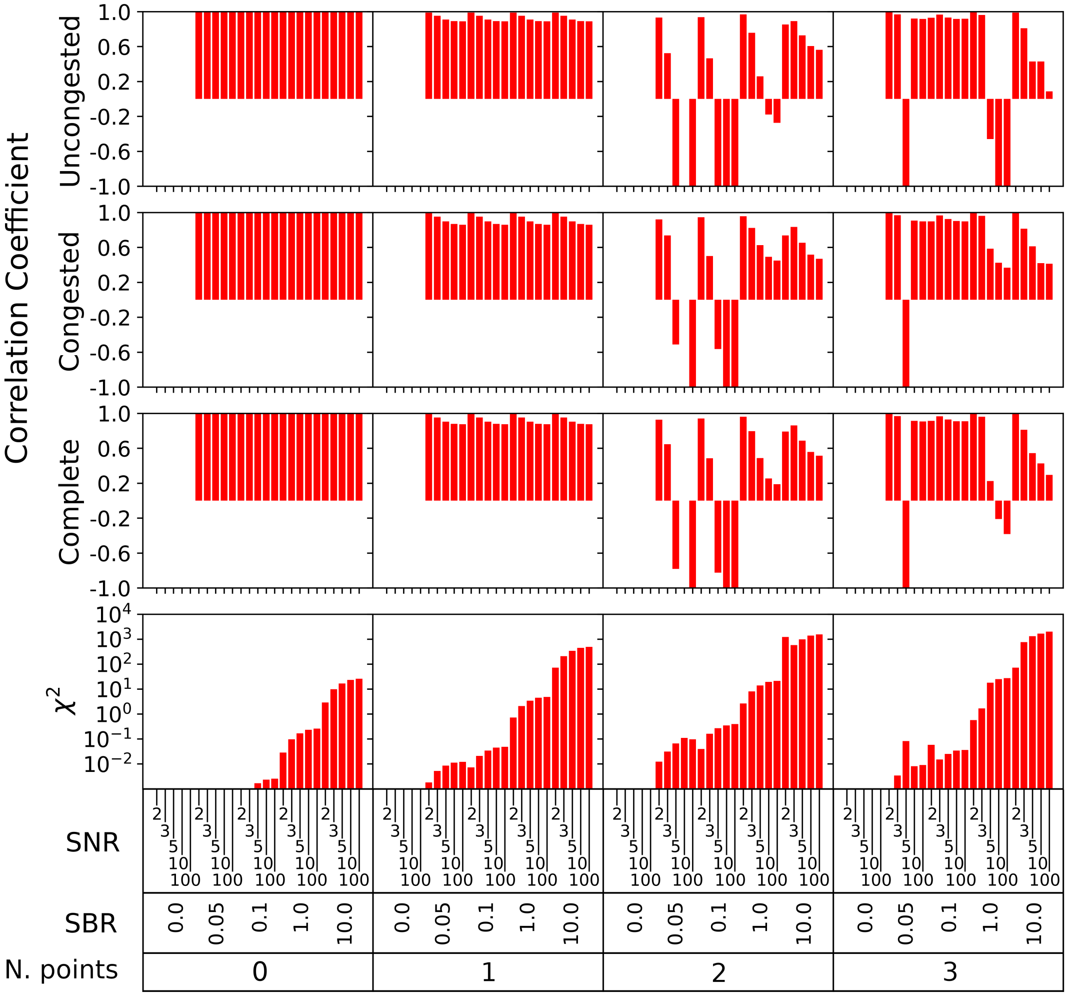

FIGURE S36 FoM reproduced according to procedure described in section 2.3 for the spectral width of 72 ppm and baseline rolling. The correlation coefficients and χ^2 are computed with respect to the spectra reconstruction instead of the baseline.

S3 | CODE LISTINGS

LISTING 1 Python code for the generation of FoM.

```
import klassez as kz
from scipy.signal import find_peaks
import scipy.signal
from scipy.stats import pearsonr

path = 'sp_4fif_fig1.acqus'

b_list = [0,  0.1, 0.3, 1.0]
SNR_theory = [2,3,5,10,100]
SNR = [[7e-2, 3.6e-2 ,1.92e-2 ,8.84e-3 ,8.313e-4], [1.38e-1, 6.34e-2 ,3.03e-2 ,1.419e-2 , 1.314e-3], [1.5e-1,
      6.62e-2 ,3.42e-2 ,1.738e-2 ,1.7574e-3], [4e-1, 1.25e-1 ,5.47e-2 ,2.278e-2 ,2.0935e-3]]

line = 3.9807192807192813

lws = np.ones(7) * 41.02240092599793  #5.7 channels
lws[0] = 71.96912443157531  #10 channels
lwG = 35.984562215787655  #5 channels
shifts = [6.618081918081918, 5.23946053946054, 4.316383616383616, 3.6930069930069935, 3.2854145854145855,
     3.0096903096903103,2.8298701298701303]

A_list = [2, 1, 1, 1, 1, 1, 1]

SBR = [0, 0.05, 0.1, 1.0, 10.0]

pSW_list = [12, 30,54,72]
TD_list = [1001, 2500, 4500,6000,]

dir = 'fig1/'

for pp,pSW in enumerate(pSW_list):

    Uncong = []
    Cong = []
    Complete = []
    chi2 = []
    Uncong_sp = []
    Cong_sp = []
    Complete_sp = []
    chi2_sp = []

    for a in SBR:

        print('\nSBR: ', a)

        chi2.append([])
        Uncong.append([])
        Cong.append([])
        Complete.append([])
        Uncong_sp.append([])
        Cong_sp.append([])
        Complete_sp.append([])
        chi2_sp.append([])

        for i in range(7):

            with open(path, 'w') as f:
                f.write('B0\t14.1\n')
                f.write('nuc\t1H\n')
                f.write('o1p\t4.7\n')
                f.write('SWp\t'+str(pSW)+'\n')
                f.write('TD\t'+str(TD_list[pp])+'\n')
                f.write('shifts\t'+str(shifts[i])+',\n')
                f.write('fwhm\t'+str(lws[i])+',\n')
                f.write('amplitudes\t'+str(A_list[i])+',\n')
                f.write('x_g\t0,\n')
                f.write('mult\ts,\n')
```

```
            f.write('Jconst\t0,\n')
            f.close()
        s = kz.Spectrum_1D(path, isexp=False)
        L = s.fid

        with open(path, 'w') as f:
            f.write('B0\t14.1\n')
            f.write('nuc\t1H\n')
            f.write('o1p\t4.7\n')
            f.write('SWp\t'+str(pSW)+'\n')
            f.write('TD\t'+str(TD_list[pp])+'\n')
            f.write('shifts\t'+str(4.7)+',\n')
            f.write('fwhm\t'+str(lwG*2.355)+',\n')
            f.write('amplitudes\t1,\n')
            f.write('x_g\t1,\n')
            f.write('mult\ts,\n')
            f.write('Jconst\t0,\n')
            f.close()
        s = kz.Spectrum_1D(path, isexp=False)
        G = s.fid

        if i==0:
            FID = L*G
        else:
            FID += L*G

    for kk,s_n in enumerate(SNR[pp]):

        chi2[-1].append([])
        Uncong[-1].append([])
        Cong[-1].append([])
        Complete[-1].append([])
        Uncong_sp[-1].append([])
        Cong_sp[-1].append([])
        Complete_sp[-1].append([])
        chi2_sp[-1].append([])

        print('SNR: ', s_n)

        for b in b_list:

            print('Baseline: ', b)

            s.fid = FID.copy()
            s.fid /= np.max(s.fid.real)
            np.random.seed(666)
            s.fid += kz.sim.noisegen(s.fid.shape, 2821, s.acqus['t1'], s_n=s_n)
            sp_nobase = kz.processing.ft(s.fid)
            sp_nobase /= np.max(sp_nobase.real)
            sp_nobase *= a*(1+b)
            s.process()

            sx,dx,_ = find_limits(10, 8, s.ppm)
            snr_r = kz.misc.snr(s.r, n_reg=(sx,dx))
            print('SNR',snr_r)

            s.S /= np.max(s.S.real)
            s.S *= a*(1+b)

            sx,dx = int(-2/500*TD_list[pp]/2), int(2/500*TD_list[pp]/2)

            sbaseline = ferf(np.linspace(sx,dx,TD_list[pp]))
            sbaseline /= np.max(sbaseline)
            sbaseline *= b
            sbaseline = (sbaseline - np.min(sbaseline))+1

            s.S += sbaseline  # introduces baseline distortion

            sp_sim = s.S

            sx,dx,_ = find_limits(10.694005994005993, -1.2940059940059925, s.ppm)

            x_sim = s.ppm
            x_freq = s.freq
```

```
line_idx = ppmfind(x_sim, line)[0]

if a!=0:
    #FIF analysis
    fif=FIF.FIF()
    fif.run(sp_sim.real)
    N = fif.data['IMC'].shape[0]

    start = 1
    stop = N

    value_list = []
    value2_list = []
    N_peaks = []
    oscill2 = []
    oscill1 = []
    baseline_prev = np.zeros_like(sp_sim.real)
    baseline_prev2 = np.zeros_like(sp_sim.real)
    peaks_indices_prev = []
    peaks_indices_prev2 = []
    for idx, Ncomp in enumerate(range(start,stop)):

        rec_spectra = np.sum([fif.data['IMC'][i,:] for i in range(0, Ncomp)], axis=0)
        baseline = np.sum([fif.data['IMC'][i,:] for i in range(Ncomp, N)], axis=0)

        oscill2.append(np.log(np.linalg.norm(grad2(baseline)))-np.log(np.linalg.norm(baseline
            )**2))
        oscill1.append(np.log(np.linalg.norm(grad1(baseline)))-np.log(np.linalg.norm(baseline
            )))

    #number of elements different from zero
    N_peaks = oscill1
    Ncomp2 = most_distant_indices(oscill2[4:-4])
    if b==0:
        Ncomp2 = most_distant_indices(oscill2[4:])
    print(Ncomp2)
    Ncomp = Ncomp2[-1]+4

    rec_spectra = np.sum([fif.data['IMC'][i,:] for i in range(0, Ncomp)], axis=0)
    baseline = np.sum([fif.data['IMC'][i,:] for i in range(Ncomp, N)], axis=0)

    fig = plt.figure()
    fig.set_size_inches(10, 2.5)
    plt.subplots_adjust(hspace=0.)
    ax = fig.add_subplot(211)
    ax.set_title(' SBR: '+str(a)+' - SNR: '+str(round(snr_r,0))+' - Slope: '+str(b))
    ax.plot(x_sim, sp_nobase.real, c='b')
    ax.plot(x_sim, rec_spectra, c='r')
    ax.set_xlim(max(x_sim), min(x_sim))
    ax.set_xticklabels([])
    ax = fig.add_subplot(212)
    ax.plot(x_sim, sbaseline, c='b')
    ax.plot(x_sim, baseline, c='r')
    ax.set_xlim(max(x_sim), min(x_sim))
    plt.savefig(dir+'slope'+str(b)+'_SNR'+str(SNR_theory[kk])+'_SBR'+str(a)+'_pSW'+str(pSW)+'
        .png', dpi=600)
    plt.close()

    chi2[-1][-1].append(np.sum((baseline[sx:dx]-sbaseline[sx:dx])**2))
    Uncong[-1][-1].append(r2_score(baseline[line_idx:dx].real, sbaseline[line_idx:dx]))
    Cong[-1][-1].append(r2_score(baseline[sx:line_idx].real, sbaseline[sx:line_idx]))
    Complete[-1][-1].append(r2_score(baseline[sx:dx].real, sbaseline[sx:dx]))
    chi2_sp[-1][-1].append(np.sum((rec_spectra[sx:dx].real-sp_nobase[sx:dx].real)**2))
    Uncong_sp[-1][-1].append(r2_score(rec_spectra[line_idx:dx].real, sp_nobase[line_idx:dx].
        real))
    Cong_sp[-1][-1].append(r2_score(rec_spectra[sx:line_idx].real, sp_nobase[sx:line_idx].
        real))
    Complete_sp[-1][-1].append(r2_score(rec_spectra[sx:dx].real, sp_nobase[sx:dx].real))


else:

    chi2[-1][-1].append(0)
```

```
                Uncong[-1][-1].append(0)
                Cong[-1][-1].append(0)
                Complete[-1][-1].append(0)
                chi2_sp[-1][-1].append(0)
                Uncong_sp[-1][-1].append(0)
                Cong_sp[-1][-1].append(0)
                Complete_sp[-1][-1].append(0)


        chi2 = np.array(chi2)
        Uncong = np.array(Uncong)
        Cong = np.array(Cong)
        Complete = np.array(Complete)
        chi2_sp = np.array(chi2_sp)
        Uncong_sp = np.array(Uncong_sp)
        Cong_sp = np.array(Cong_sp)
        Complete_sp = np.array(Complete_sp)

        # ... figure generation ...
```

LISTING

2 Python code for the correction of synthetic baseline distorted monodimensional spectra using FIF algorithm (see Figure 1).

```
import klassez as kz
import numpy as np
import matplotlib.pyplot as plt
import FIF_main as FIF

path = 'sp_4fif.acqus'

lws = np.ones(4) * 50

b = 2  # number of altered points (N. points)

s = kz.Spectrum_1D(path, isexp=False)
s.fid += kz.sim.noisegen(s.fid.shape, 3000, s.acqus['t1'], s_n=0.6)
sp_nobase = kz.processing.ft(s.fid)

s.fid[:b] += np.ones_like(s.fid[:b])*100  # introduces baseline distortion
s.process()

sp_sim = s.S
x_sim = s.ppm

#FIF analysis
fif=FIF.FIF()
fif.run(sp_sim.real)
N = fif.data['IMC'].shape[0]

sim_spectra = np.zeros(len(sp_sim))
for j in range(N):
    sim_spectra += fif.data['IMC'][j,:].real
print('ERROR: ', np.sum(np.abs(sim_spectra-sp_sim.real)))

fig, ax = plt.subplots(1, 1, figsize=(5, 4))
ax.plot(x_sim, sp_sim.real, label='original spectrum', c='black', lw=0.8)
ax.set_xlabel('frequency scale / ppm')
ax.set_ylabel('Intensity / a.u.')
ax.set_xlim(20,-20)
plt.tight_layout()
plt.savefig('fig1_sp'+str(b)+'.png', dpi=600)
plt.show()

## one of the subplots
rec_spectra = np.sum([fif.data['IMC'][i,:] for i in range(0, 2)], axis=0)

fig, ax = plt.subplots(1, 1, figsize=(7, 1.6))
ax.plot(x_sim, rec_spectra, c='black', lw=0.8, label='sum IMFs 1-2')
ax.set_xlim(20,-20)
ax.tick_params(axis='both', labelsize=14)
```

```
ax.set_xlabel('frequency scale / ppm', fontsize=14)
plt.legend(fontsize=14, loc='upper right')
plt.tight_layout()
plt.savefig('fig2_sp'+str(b)+'.png', dpi=600)
plt.show()
```

LISTING 3 Implementation of code for generation of Figure 2.

```
import klassez as kz
import numpy as np
import matplotlib.pyplot as plt
import FIF_main as FIF

path = 'sp_4fif.acqus'

lws = np.ones(4) * 50

b = 2
Ncomp = 44   # choice of the number of components for spectra reconstruction
             # the remaining components are considered as baseline

s = kz.Spectrum_1D(path, isexp=False)
s.fid += kz.sim.noisegen(s.fid.shape, 3000, s.acqus['t1'], s_n=0.3)
sp_nobase = kz.processing.ft(s.fid)

s.fid[:b] += np.ones_like(s.fid[:b])*100  # introduces baseline distortion
s.process()

sp_sim = s.S
x_sim = s.ppm

#FIF analysis
fif=FIF.FIF()
fif.run(sp_sim.real)
N = fif.data['IMC'].shape[0]

sim_spectra = np.zeros(len(sp_sim))
for j in range(N):
    sim_spectra += fif.data['IMC'][j,:].real
print('ERROR: ', np.sum(np.abs(sim_spectra-sp_sim.real)))
print('RMSD: ', np.sqrt(np.mean((sim_spectra-sp_sim.real)**2)))

rec_spectra = np.sum([fif.data['IMC'][i,:] for i in range(0, Ncomp)], axis=0)
baseline = np.sum([fif.data['IMC'][i,:] for i in range(Ncomp, N)], axis=0)

fig, ax = plt.subplots(1, 1, figsize=(5, 4))
ax.plot(x_sim, sp_sim.real, label='original spectrum', c='magenta', lw=0.8)
ax.plot(x_sim, rec_spectra, label=f'{Ncomp} IMFs out of {N}', c='green', lw=0.8)
ax.plot(x_sim, baseline, label='baseline', c='blue', lw=0.8)
ax.axhline(0,linestyle='--', color='grey', lw=0.5)
ax.set_xlabel('frequency scale / ppm')
ax.set_ylabel('Intensity / a.u.')
ax.set_xlim(20,-20)
plt.tight_layout()
plt.legend()
plt.show()
```

LISTING 4 Functions used in listings 5 and 6 to read experimental spectra, perform direct and inverse FT, and apply windowing function.

```
def nmr_spectra_1d(path):

    phases = np.zeros(2)
    phases[0] = 0#input('Phase 0: ')
    phases[1] = 0#input('Phase 1: ')

    dic, datare = ng.bruker.read_pdata(path, bin_files=['1r'])
```

```
    dic, dataim = ng.bruker.read_pdata(path, bin_files=['1i'])
    data = datare + 1j*dataim      #recombine Re and Im to get the complete complex data

    udic = ng.bruker.guess_udic(dic, data)
    C = ng.convert.converter()
    C.from_bruker(dic, data, udic)
    dicpipe, datapipe = C.to_pipe()
    uc = ng.pipe.make_uc(dicpipe, datapipe)
    ppm_scale = uc.ppm_scale()

    def phaseproc(dic, data, ph0=0, ph1=0):
        dicp, datap = ng.pipe_proc.ps(dic, data, p0=ph0, p1=ph1)
        return dicp, datap

    dicp, datap = phaseproc(dicpipe, datapipe, ph0 = phases[0], ph1 = phases[1])   #perform phase correction

    return datap, ppm_scale, dic

def ft(fid, td = 65536, dw = 1e-6, o1p = 4.7, sfo1 = 1200):
    data = np.zeros_like(fid)
    fid[0] /= 2
    data = np.fft.fftshift(np.fft.fft(fid))

    freq = np.zeros_like(fid)
    freq = np.fft.fftshift(np.fft.fftfreq(td, d=dw)) / sfo1 + o1p
    return data, freq

def ift(data):
    fid = np.zeros_like(data)
    fid = np.fft.ifft(np.fft.ifftshift(data))
    fid[0] *= 2
    return fid

def windowing_sig(data, L=None, subtract_mean=False):

    """
    Applies a smooth windowing function on data.
    Let data be a 1darray of N points, and let x integer.
    Then, the windowing function is a piecewise-function defined as follows:

        { 1/2 [ cos( x * pi/L - pi) + 1 ]   with 0 <= x <= L     for i = 0,...,L-1
    f = { 1                                                      for i = L-1,...,N-L-1
        { 1/2 [ cos(-x * pi/L - pi) + 1 ]   with -L <= x <= 0    for i = N-L,...,N-1
    ------------------
    Parameters:
    - data: 1darray
        Data to be windowe
    - L: int
        Length of the wings,in points. Default value: N//2
    - subtract_mean: bool
        If True, subtracts the mean of data before to apply f, then adds it afterwards.
    ------------------
    Returns:
    - s_w: 1darray
        Windowed data
    """
    # Determine default value of L
    if L is None:   # Half of data length
        L = data.shape[-1] // 2
    else:           # Make sure it is integer
        L = int(L)

    # Shallow copy of data
    s = np.copy(data)
    # Length of data
    Ls = s.shape[-1]
    # Check if it is possible to compute this function
    assert Ls >= 2*L, 'The length of the wings cannot be longer than the data itself!'

    # If you want to subtract the mean of the data before to apply the windowing
    if subtract_mean:
        u = np.mean(s)
    else:
        u = 0
```

```
    # Dummy values for computing the wings
    x = np.arange(L)
    # Left wing: cosine function which is 0 at index 0 and 1 at index L
    l_side = 0.5 * (np.cos(x * np.pi/L - np.pi) + 1)
    # The right wing is the mirror image of the left wing
    r_side = l_side[::-1]
    # Flat region of 1 between the wings
    ones = np.ones(Ls - 2*L)
    # Glue everything together
    f = np.concatenate([l_side, ones, r_side], axis=-1)

    # Apply windowing on s
    s_w = (s - u) * f + u

    return s_w
```

LISTING 5 Implementation of code for generation of Figures 3 and 4.

```
import klassez as kz
import numpy as np
import matplotlib.pyplot as plt
import FIF_main as FIF

path='NiSAL_HDPT_200929/10/pdata/10'

datap, x, ngdicp = nmr_spectra_1d(path)

fid = ift(datap)
dw = 1/(ngdicp['acqus']['SW_h'])
td = int(ngdicp['acqus']['TD'])//2
o1 = ngdicp['acqus']['O1']
sf1 = ngdicp['acqus']['SFO1']
o1p = o1/sf1
datap = ft(fid, td, dw, o1p, sf1)[0]

y = datap.real

Ncomp = 48

fif=FIF.FIF()
fif.run(y)

N = fif.data['IMC'].shape[0]

#plot the decomposition
#figure_decomp(x, y, fif)

rec_spectra = np.sum([fif.data['IMC'][i,:] for i in range(0, Ncomp)], axis=0)
baseline = np.sum([fif.data['IMC'][i,:] for i in range(Ncomp, N)], axis=0)

fig, ax = plt.subplots(1, 1, figsize=(6, 4))
ax.plot(x, y.real, label='original spectrum', c='magenta', lw=0.8)
ax.plot(x, rec_spectra, label=f'{Ncomp} IMFs out of {N}', c='green', lw=0.8)
ax.plot(x, baseline, label='baseline', c='blue', lw=0.8)
ax.axhline(0,linestyle='--', color='grey', lw=0.5)
ax.set_xlabel(r'$\delta ^1$H  / ppm', fontsize=12)
ax.set_ylabel('Intensity / a.u.', fontsize=12)
plt.xlim(470, -370)
plt.ylim(-4e12, 1.15e13)
plt.tight_layout()
plt.legend()
plt.show()
```

LISTING 6 Implementation of code for generation of Figure 5.

```
import klassez as kz
```

```
import numpy as np
import matplotlib.pyplot as plt
import FIF_main as FIF

path = '19F_testsinglefid_150323/1/pdata/1'

datap, x, ngdicp = nmr_spectra_1d(path)

fid = ift(datap)
dw = 1/(ngdicp['acqus']['SW_h'])
td = int(ngdicp['acqus']['TD'])//2
o1 = ngdicp['acqus']['O1']
sf1 = ngdicp['acqus']['SFO1']
o1p = o1/sf1
datap = ft(fid, td, dw, o1p, sf1)[0]

y = datap.real

y0 = np.copy(y)
y = windowing_sig(y, L=1/6*len(y), subtract_mean=True)

Ncomp = 40

fif=FIF.FIF()
fif.run(y)

N = fif.data['IMC'].shape[0]

baseline = np.sum([fif.data['IMC'][i,:] for i in range(Ncomp, N)], axis=0)
rec_spectra = np.sum([fif.data['IMC'][i,:] for i in range(0, Ncomp)], axis=0)
baseline = np.reshape(baseline, (1,len(baseline)))
rec_spectra = np.reshape(rec_spectra, (1, len(rec_spectra)))

fig, ax = plt.subplots(1, 1, figsize=(6.5, 5))
ax.plot(x, y0, label = "original spectra", c="red")
ax.plot(x, baseline[0,:], label = "baseline", c="blue")
ax.plot(x, rec_spectra[0,:], label = "reconstructed signal", c="green")
ax.plot(x, y, '--',label = "original spectra * wf", c="magenta")
plt.xlim(max(x), min(x))
plt.xticks(fontsize=12)
plt.yticks(fontsize=12)
plt.ylabel('Intensity (a.u.)', fontsize=12)
plt.xlabel(r'$\delta {^{19}}F (ppm)$', fontsize=12)
plt.legend(fontsize=12)
plt.tight_layout()
plt.show()
plt.close()
```
